# Heritability of the human connectome: A connectotyping study

**DOI:** 10.1162/netn_a_00029

**Published:** 2018-06-01

**Authors:** Oscar Miranda-Dominguez, Eric Feczko, David S. Grayson, Hasse Walum, Joel T. Nigg, Damien A. Fair

**Affiliations:** Department of Behavioral Neuroscience, Oregon Health and Science University, Portland, OR, USA; Department of Behavioral Neuroscience, Oregon Health and Science University, Portland, OR, USA; Department of Behavioral Neuroscience, Oregon Health and Science University, Portland, OR, USA; Center for Neuroscience, University of California, Davis, Davis, CA, USA; Silvio O. Conte Center for Oxytocin and Social Cognition, Center for Translational Social Neuroscience, Yerkes National Primate Research Center, Department of Psychiatry and Behavioral Sciences, Emory University, Atlanta, GA, USA; Department of Behavioral Neuroscience, Oregon Health and Science University, Portland, OR, USA; Department of Psychiatry, Oregon Health and Science University, Portland, OR, USA; Department of Behavioral Neuroscience, Oregon Health and Science University, Portland, OR, USA; Department of Psychiatry, Oregon Health and Science University, Portland, OR, USA; Advanced Imaging Research Center, Oregon Health and Science University, Portland, OR, USA

**Keywords:** Development, Heritability, Effective connectivity, MRI, Functional connectivity, Resting-state MRI

## Abstract

Recent progress in resting-state neuroimaging demonstrates that the brain exhibits highly individualized patterns of functional connectivity—a “connectotype.” How these individualized patterns may be constrained by environment and genetics is unknown. Here we ask whether the connectotype is familial and heritable. Using a novel approach to estimate familiality via a machine-learning framework, we analyzed resting-state fMRI scans from two well-characterized samples of child and adult siblings. First we show that individual connectotypes were reliably identified even several years after the initial scanning timepoint. Familial relationships between participants, such as siblings versus those who are unrelated, were also accurately characterized. The connectotype demonstrated substantial heritability driven by high-order systems including the fronto-parietal, dorsal attention, ventral attention, cingulo-opercular, and default systems. This work suggests that shared genetics and environment contribute toward producing complex, individualized patterns of distributed brain activity, rather than constraining local aspects of function. These insights offer new strategies for characterizing individual aberrations in brain function and evaluating heritability of brain networks.

## Author Summary

By using machine learning and two independent datasets, this report shows that the brain’s individualized functional connectome or connectotype is familial and heritable. First we expand previous findings showing that by using a model-based approach to characterize functional connectivity, we can reliably identify and track *individual* brain signatures—a functional “fingerprint” or “connectotype” for the human brain—in both children and adults. Such signatures can also be used to characterize familial and heritable patterns of brain connectivity, even using limited data. Most heritable systems include the fronto-parietal, dorsal attention, ventral attention, cingulo-opercular, and default systems. Our proposed approach offers new strategies for characterizing normative development as well as altered patterns of brain connectivity and assists in characterizing the associations between genetic and epigenetic factors with brain function.

## INTRODUCTION

One of the most promising methodologies for identifying typical and atypical features of brain function is resting-state functional magnetic resonance imaging (rs-fMRI). Rs-fMRI measures spontaneously correlated activity, termed functional connectivity (FC), between pairs of regions across the brain (i.e., the functional connectome).Common versus distinct aspects of the brain’s functional connectome between individuals is an emerging focus of current research efforts (Van Essen et al., 2013). Thus, investigations that probe how variance in the human connectome is shared among relatives (i.e., heritability, which implies shared genetics, or familiality, which we define here as similarities due to shared genetics and/or shared environment) are critical for understanding how genetics and environment collectively shape common and unique patterns of brain organization. Furthermore, current trends in neuropsychiatric imaging research emphasize the importance of identifying individualized patterns of brain FC (Laumann et al., 2015; Miranda-Dominguez, Mills, Carpenter, et al., 2014) that may relate to complex genetic and environmental factors.

In a recent report we used rs-fMRI to characterize functional brain organization in individuals. In this work the activity in each brain region was modeled as the weighed combination of the remaining brain areas—a so-called connectotype, or functional fingerprint of an individual (Miranda-Dominguez, Mills, Carpenter, et al., 2014). The connectotype corresponds to a personalized connectivity matrix composed of the unique functional relationships across all brain regions. Similar to other work using fMRI (Finn et al., 2015; Poldrack et al., 2013), our approach accurately classifies the same individual at a later date. Further, this model is effective even with short acquisition times (Laumann et al., 2015; Miranda-Dominguez, Mills, Carpenter, et al., 2014).The approach is therefore well suited to study the uniqueness, familiality, and heritability of the functional connectome.

This prior work revealed meaningful shared variance between individual unrelated adults throughout the brain’s multiple functional systems, indicating that functional brain organization is largely similar among healthy adults. In contrast, the capacity to classify an individual adult was driven by a specific set of connections in higher order heteromodal association areas in frontal and parietal cortices (Miranda-Dominguez, Mills, Carpenter, et al., 2014). These regions are phylogenetically late-developing regions that are disproportionately enlarged in humans relative to other primates (Buckner & Krienen, 2013; Mueller et al., 2013), suggesting that functional circuits formed by such regions may have a dominant role in characterizing individual differences (Mueller et al., 2013).

The current study extends that prior work by examining (a) stability of the connectotype during development and (b) similarities of the connectotype between siblings and twins. Importantly, in the era of scientific rigor and reproducibility (Collins & Tabak, 2014), we maximize our understanding of the generalizability of the current findings, models, and data, by taking advantage of a secondary validation dataset.

Thus far, few studies have examined the familiality or heritability of the connectome. Structural MRI findings in adults show strong evidence of genetic and environmental influences on structural properties of the brain such as volume, shape, and white matter integrity (Blokland, de Zubicaray, McMahon, & Wright, 2012). Interestingly, and pertinent to our work here, this genetic influence seems to be modulated by age (Batouli, Trollor, Wen, & Sachdev, 2014; Schmitt et al., 2014), underscoring the importance of developmental timing.

There is mixed evidence for heritability in evoked fMRI responses (Moodie, Wisner, & MacDonald, 2014) and in rs-fMRI (Glahn et al., 2010; Korgaonkar, Ram, Williams, Gatt, & Grieve, 2014). Most findings imply that FC of the whole brain is heritable, but some data suggest heritability estimates may depend on how the rs-fMRI time courses were filtered (Fornito et al., 2011). Studies also suggest specific global network properties (Sinclair et al., 2015; van den Heuvel et al., 2013) and connectivity properties of specific brain systems (Fu et al., 2015; Yang et al., 2016) show some form of heritability. With that said, the heritability of complex, distributed activity in large samples and with strict control of structural confounds has not been evaluated across a fully parcellated and interconnected schema of brain connectivity. Furthermore, prior methods of testing heritability (Visscher & Goddard, 2015) have generally involved univariate or bivariate approaches and have not tested heritability estimates using independent data. By evaluating similarities in the connectotype between familial and nonfamilial pairs of subjects, this report builds upon prior work and provides an approach to examine familial and heritable aspects of the functional connectome.

We first examine the stability of connectotypes in youth over a 1- to 2-year span to test whether individual aspects of functional brain architecture persist despite typical developmental changes. We then use a machine-learning approach to test familiality (or similarities in the connectome due to shared genetics and/or shared environment) by examining whether connectotyping can distinguish sibling pairs (including twins) from unrelated pairs of participants, first in children, then in adults, and last, across datasets. To control for the possibility that our results reflect underlying anatomical variation, we further test the same types of models using anatomical features. Finally, we evaluate the evidence for heritability (i.e., shared genetics) of the connectotype using more traditional statistical methods.

## RESULTS

### Comparing the Consistency of the Connectotype Within Individuals, Between Siblings, and Across Unrelated Pairs

The first goals of this study were (a) to test whether rs-fMRI scans demonstrate unique signatures that persist over development, and (b) to test whether these individualized signatures are more similar between siblings versus unrelated participants. We used the connectotype as a model for each individual (Miranda-Dominguez, Mills, Carpenter, et al., 2014), composed of the coefficients of a multiple linear regression between a given region of interest (ROI) time course and all other ROIs. This procedure forms a matrix of the functional relationships between each pair of ROIs in the whole-brain network. Unlike associative measures often used in conventional rs-fMRI analyses (Fox, Zhang, Snyder, & Raichle, 2009), the connectotype coefficients signify the unique and directed contributions that each ROI makes toward activity in each other ROI. Ultimately, this method allows each ROI time course to be predicted as the weighted sum of all the others. Previously we showed that by looking at the correlation coefficient between predicted and observed time courses we can determine whether the scans used for modeling and prediction came from the same individual, even if the scans were acquired 2 weeks later (Miranda-Dominguez, Mills, Carpenter, et al., 2014). Using a longitudinal sample, here we extended this time frame to 1–2 years, and also asked whether siblings had more similar connectotype-based predictions versus unrelated participants. This longitudinal sample of youths (*N* = 188 scans) consists of 159 participants, where 131 participants have 1 scan, 27 participants have 2 scans, and 1 participant has 3 scans, totaling 188 unique scans. The sample has 16 unique sibling pairs scanned multiple times (Supplementary Table S1, Miranda-Dominguez et al., 2018), yielding a total of 46 sibling scan-pairs. (Note: Because the simple Pearson’s correlation is the most established and widely used measure of functional connectivity, we also repeat the same comparisons for this section and all the other sections of the manuscript in the Supplementary Information [Miranda-Dominguez et al., 2018] using Pearson correlation matrices for functional connectivity.)

**Figure 1.**
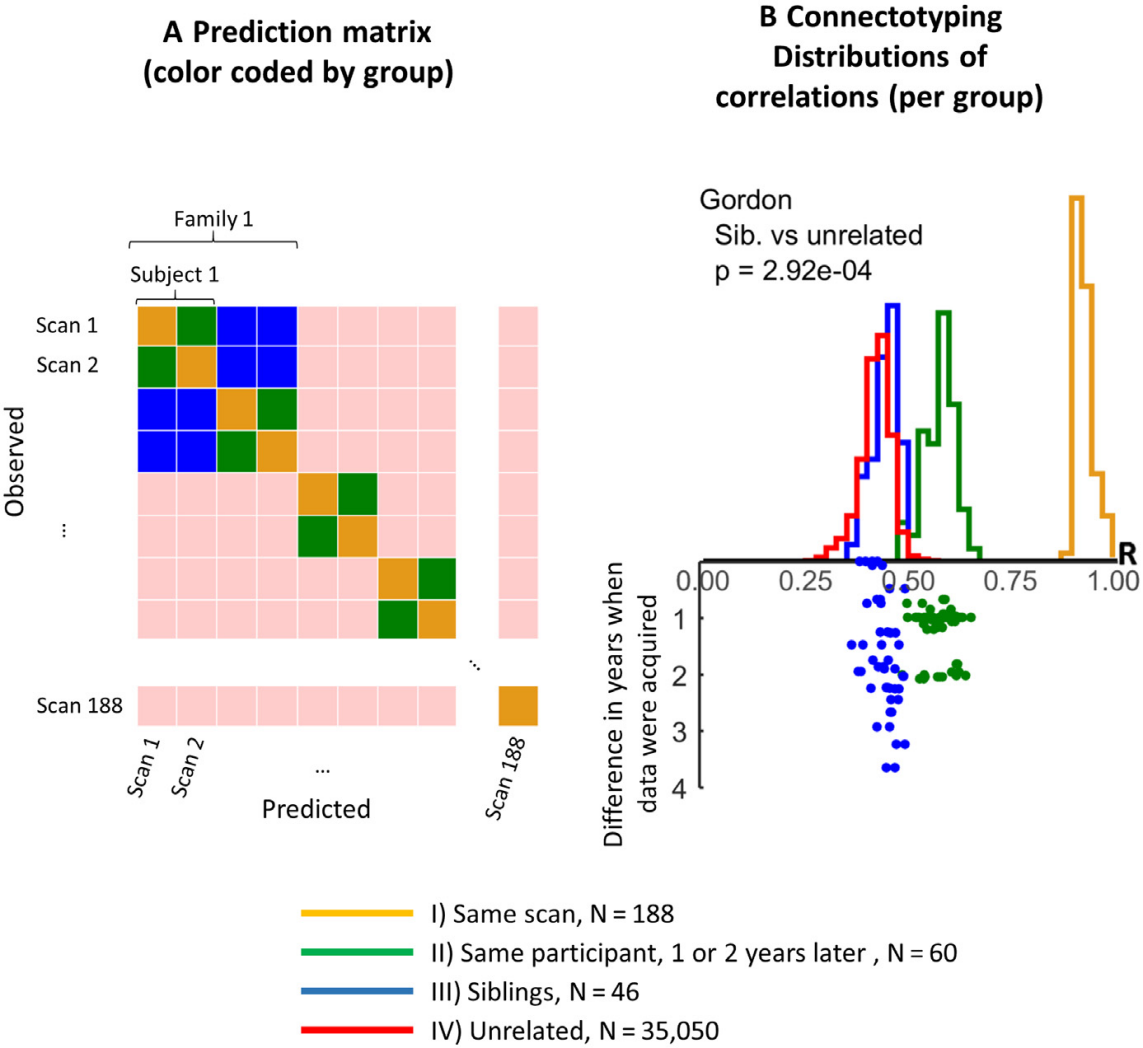
Segregating groups of paired data (same scan, same participant, siblings, and unrelated). (A) A subsection of the 188 × 188 scan-pairs being compared; the relationship between the subjects each scan comes from is color coded as indicated in the legend at the bottom. (B) The distributions of average correlation coefficients between the predicted and observed time courses across all the paired comparisons, and (bottom) the differences in age between each paired data.

From here, we compared each unique scan-pair (self-inclusive) via connectotyping (Miranda-Dominguez, Mills, Carpenter, et al., 2014; see Figure 1, and Figure 1-Figure Supplement 1 in the Supplementary Information, Miranda-Dominguez et al., 2018). To do this, we used time courses (after removing autocorrelations) from each scan to calculate a connectotype matrix of size nROI × nROI (i.e., to calculate a model), where nROI is the number of ROIs. This model was used to predict the time courses from each available scan. Predictions were calculated by multiplying the connectotype matrix obtained from each scan by the time courses from each scan (ROI × number of available frames). When a scan was used to predict its own time courses (yellow histogram in Figure 1), the given scan’s time courses were partitioned into two independent samples, where one sample was used for connectotyping and the second one for prediction (i.e., we always used fresh data). Given the 188 scans, the total number of comparisons (*N* = 188 × 188 = 35,344 predictions) consists of the following (Figure 1):

I)  Same individual, predicting data in same scan session, *N* = 188;
II)  Same individual, predicting data in a different scan (1 or 2 years later), *N* = 60;
III)  Individual predicting a sibling, *N* = 46;
IV)  Individual predicting an unrelated individual, *N* = 35,050.

**Figure 2.**
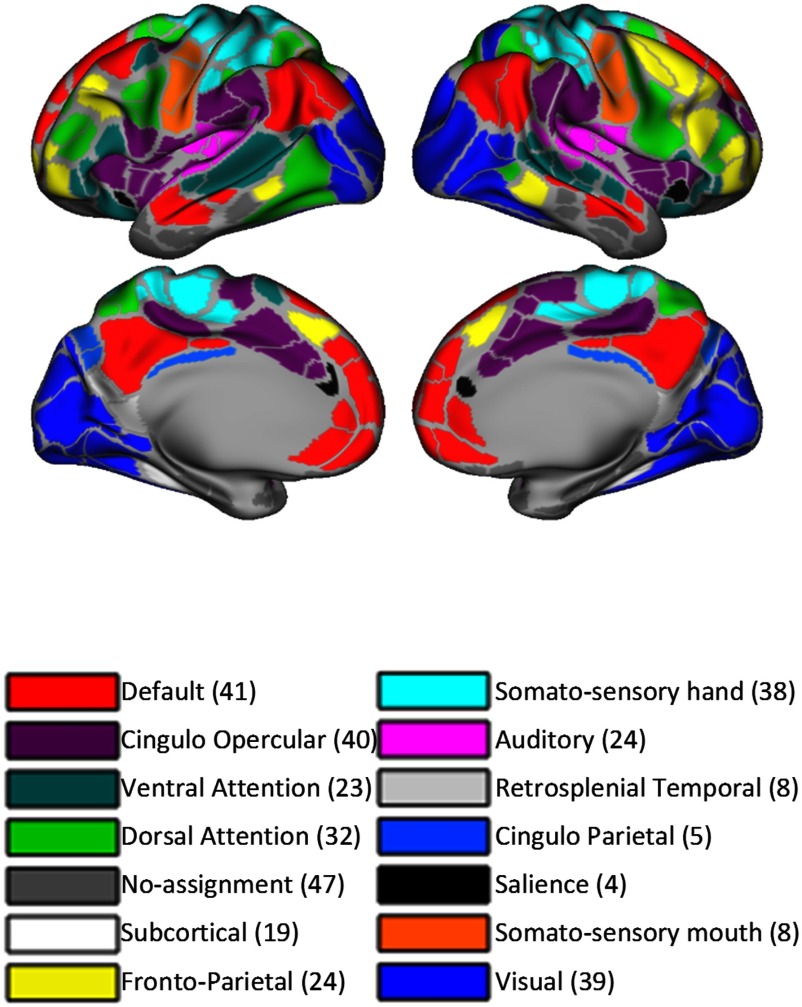
Gordon parcellation and ROIs per network. This cartoon shows the respective location of each functional network on the brain’s surface as well as the number of ROIs per network, as described in Gordon et al. (2014).

For each comparison in the four groups, we calculated the correlation coefficient between predicted and observed time courses, ending up with as many correlations as ROIs for each scan-pair being compared. Figure 2 shows the data-driven ROI set proposed by Gordon et al. (2014). These correlations (*R*) were then averaged providing one mean correlation value for each paired comparison. The distributions across all pairs are shown in Figure 1B. The data show that connectotyping uniquely identified the same individuals predicting fresh data from the same scan session (Group I, average *R* > 0.8). The data also show that connectotyping can predict the same individual in a different scan session, even when the data are acquired up to 2 years apart (Group II, average *R* = 0.58). Interestingly, given this sample we find no evidence of any association between the years between scan sessions and predictability (Figure 1- Figure Supplement 2, Miranda-Dominguez et al., 2018). The correlation coefficient between the average similarities between scans and time between scans was *R* = 0.0079 (*p* = 0.95) for Group II (Figure 1-Figure Supplement 2, Miranda-Dominguez et al., 2018).

Furthermore, predictions among siblings (Group III, average *R* = 0.44) compared with an individual predicting an unrelated participant (Group IV, average *R* = 0.42) are statistically different (*t* test comparing the distributions of predictions between siblings versus predictions among unrelated participants after applying Fisher Z transformation of each correlation value, *p* = 2.56 × 10^−4^). To note, while five parcellation schemes were examined (Figure 1-Figure Supplement 3, Miranda-Dominguez et al., 2018)—Gordon (Gordon et al., 2014), HCP (Glasser et al., 2016), Power (Power et al., 2011), Yeo (Yeo et al., 2011), and Markov (Markov et al., 2014; Miranda-Dominguez, Mills, Grayson, 2014)—we ran all subsequent analyses using the Gordon parcellation (Figure 2). We did so because it slightly outperformed most of the others in distinguishing siblings from unrelated groups (*t* test comparing the distributions of predictions between siblings versus predictions among unrelated participants after applying Fisher Z transformation of each correlation value: *p* values are 2.56 × 10^−4^, 1.96 × 10^−4^, 8.84 × 10^−4^, 9.24 × 10^−3^, and 2.99 × 10^−2^, for Gordon, HCP, Power, Yeo, and Markov parcellations, respectively; see Figure 1-Figure Supplement 3, Miranda-Dominguez et al., 2018), and also because this parcellation schema, both delineates the full boundary of each brain region and reports the belonging of each ROI into functional systems.

We should note that the lack of large effects between siblings versus unrelated participants found above suggests that a more refined analysis is needed, where individual or subsets of ROIs can drive effects as opposed to one ROI at a time. This approach may be a better test of whether connectotyping is more similar between familial than nonfamilial pairs. In the next section, we conduct such a test via multivariate statistical models and further explore which ROIs may better differentiate siblings from unrelated pairs.

In summary, connectotyping captures individual functional brain signatures that persist across developmental time spans (1–2 years). In addition, the connectotype is influenced by family status.

### Classification of Sibling Pairs in Youth

We next sought to determine what specific patterns of brain connectivity are shared in families, and how well sibling pairs can be distinguished (i.e., classified) from unrelated pairs using multivariate statistics. Here we used longitudinal data coming from 16 unique sibling pairs scanned multiple times, yielding a total of 46 sibling scan-pairs, and 35,050 unrelated comparisons (Supplementary Table S1, Miranda-Dominguez et al., 2018). We approached this analysis using support vector machine (SVM) and trained *N* = 1,000 SVM classifiers, randomly selecting and reserving for each run three of the sibling scan-pairs for prediction (using the remaining 43 for training), along with matched samples of randomly selected unrelated scan-pairs. Classifiers were trained to detect whether the scan-pairs came from siblings or unrelated individuals. In this analysis, the same siblings from one scanning timepoint could be included in the training dataset and at another timepoint be used to test the prediction accuracy.

**Figure 3.**
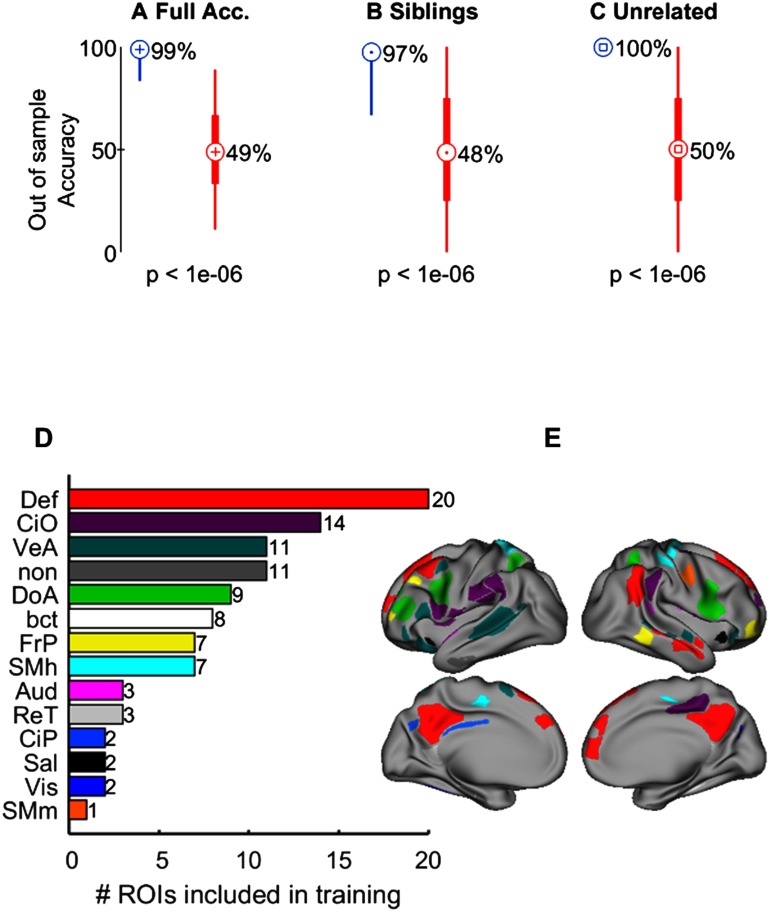
Classifying siblings versus unrelated populations when other sibling pairs of the same family are included in the training set (sample of youths). (A) The distributions of out-of-sample accuracy (*N* = 1,000) when predicting siblings and unrelated participants (blue) versus the null hypothesis (red), which was obtained by running the same classifiers (*N* = 1,000) with the same features but randomizing the labels (i.e., sibling or unrelated). Each distribution highlights the percentiles 2.5 and 97.5 with a thin line. Thick lines are used to highlight the percentiles 25 and 75, while the central markers are used to show the mean values. (B) The out-of-sample accuracies when predicting only siblings. (C) The out-of-sample accuracies when predicting only unrelated participants. (D) The consensus’ ROIs per functional network (as defined by Gordon et al., 2014) used in the classifier. (E) The location of such ROIs on the surface of the brain.

Features in the SVM were defined as the correlation coefficient between the observed and the predicted time courses for each ROI. A subset comprising 100 of the Gordon ROIs were selected as the features for classification by obtaining the features with the largest between-group differences (siblings versus unrelated individuals), assessed via the Kolmogorov-Smirnov test (see Supplementary Table S2, Miranda-Dominguez et al., 2018, for a list of all the ROIs sorted according to their rank in differentiating siblings from unrelated participants). We found that the classifiers had, on average, an out-of-sample accuracy of 99%, with a sensitivity of 97% and a specificity of 100% (*p* < 10^−6^ for all, Figure 3A–C). The classification was driven by the following functional networks: default, cingulo-opercular, ventral-attention, dorsal attention, and fronto-parietal, (Figure 3D–E; Figure 3-Figure Supplement 1, Miranda-Dominguez et al., 2018).

For further clarification of the findings, the 11 ROIs (out of 47) with no assignment to a functional network that were among the top 100 ROIs (Supplementary Table S2, Miranda-Dominguez et al., 2018) were also mapped into the appropriate communities defined by Yeo et al. (2011) using a “winner-take-all” approach. These data are shown in Supplementary Table S3 and Figure 3-Figure Supplement 2 (Miranda-Dominguez et al., 2018). It can be seen that most of these 11 ROIs partially belong to multiple networks: default, limbic, or fronto-parietal. Following a winner-take-all approach, three ROIs end up in the default and seven in the limbic system, and one ROI remains unclassified.

To test whether the overall findings were driven by differences in terms of ROI size, we did a Kolmogorov-Smirnov test to compare the ROI size (number of grayordinates) of the cortical features used for classification versus the size of the excluded ROIs, being *k* = 0.1169, *p* = 0.3041, suggesting no evidence of differences in terms of ROI size between samples (see Figure 3-Figure Supplement 3, Miranda-Dominguez et al., 2018).

To determine the robustness of this approach with regard to the number of features, we repeated this analysis using the top 20, 40, … until including all the features in the training set, finding no differences in classification accuracy when at least 60 features are included. (Figure 3-Figure Supplement 4A shows the corresponding results and Supplementary Table S2 lists the Gordon ROIs sorted according to their rank in differentiating siblings from unrelated participants, including size and the functional network each ROI belongs to; see Miranda-Dominguez et al., 2018.)

Interestingly, if the classifiers are trained using only ROIs within each functional network, the dorsal and ventral attention, cingulo-opercular, fronto-parietal, and default mode networks provide the highest accuracy (Figure 3-Figure Supplement 4B, Miranda-Dominguez et al., 2018). Importantly, the familality of motion was also quantified to rule out shared family characteristics of motion to the current data. Motion could not account for the current findings (see Supplementary Information, Miranda-Dominguez et al., 2018). These data demonstrate that shared patterns of brain FC, particularly in higher order heteromodal systems, can accurately identify familial relationships between participants.

While these results suggest that familial brain connectivity patterns are generalizable to some extent, we retested whether familial relationships could be classified more accurately by applying the same methods in a larger sample taken from the Human Connectome Project (HCP).

### Classification of Sibling Pairs Using Connectotyping in Adults Using Human Connectome Project Data

To test the generalizability of familial patterns of brain connectivity, we used a subset of 198 scans coming from 59 unique families (Supplementary Tables S4 and S5, Miranda-Dominguez et al., 2018), although we also ran models on the full 499 sample, obtaining similar levels of out-of-sample accuracies, as shown in Figure 4-Figure Supplement 1 (Miranda-Dominguez et al., 2018); see the Methods section for details.

**Figure 4.**
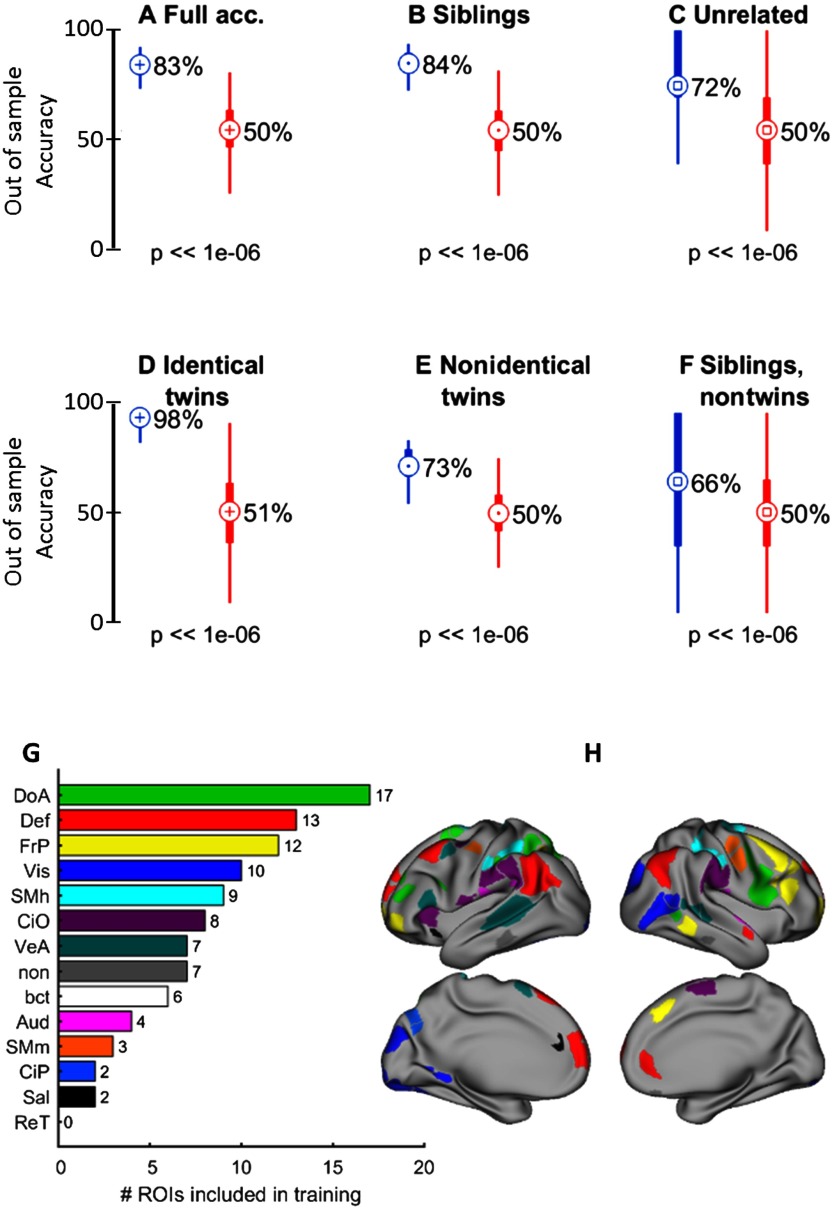
Classifying siblings versus unrelated populations, when families with twins and other sibling pairs of the same family were not included in the training set (HCP dataset). (A–F) show the distributions of (A) full accuracy, (B) siblings, (C) unrelated, (D) monozygotic, (E) dizygotic, and (F) nontwin sibling pairs for the connectotyping-based SVM classifier versus the results obtained when the same classifiers were run after randomizing the labels (sibling or unrelated). (G) The consensus’ distribution of ROIs per functional network used in the classifier. (H) The location of such ROIs on the surface of the brain.

On each run (*N* = 1,000), three families were randomly selected and reserved for prediction, leaving the remaining families for training (matching each partition with unrelated pairs). Importantly, only families with no twins were used for training. Again, we selected on each run the top 100 features that were most distinct between siblings and unrelated pairs (data from the participants assigned to the partition “prediction” were not used to rank features). In addition to predicting siblings versus unrelated participants, we also used the classifiers to predict twins. In this case, the out-of-sample accuracy was 83%, with a sensitivity of 84% and a specificity of 72% (*p* < 10^−6^, *t* test comparing each distribution versus null models obtained using the same approach but randomizing the labels, i.e., sibling or unrelated) for all (Figure 4A–C). Interestingly, the accuracy was higher for identical twins (98%) than for nonidentical twins (73%; significance of difference: *p* < 10^−6^ rank-sum test, Cohen’s *d* = 3.38) and nontwin siblings (72%; *p* < 10^−6^ for all as shown in Figure 4D–F), which is a significant finding, since families with twins were excluded from training. The classifications were mainly driven by ROIs belonging to the following functional networks: dorsal attention, default mode, fronto-parietal, visual, somatosensory hand, cingulo-opercular, and ventral attention (Figure 4G–H). As before, we did not notice significant change in accuracy if a different number of features were selected when at least 60 features were included (Figure 4-Figure Supplement 2, Miranda-Dominguez et al., 2018).

Overall, these results suggest that kinship substantially contributes to individualized patterns of complex brain organization. Furthermore, greater accuracy for predicting monozygotic versus dizygotic twins strongly suggests that these patterns are partially heritable.

### Classification of Sibling Pairs Using Independent Datasets

**Figure 5.**
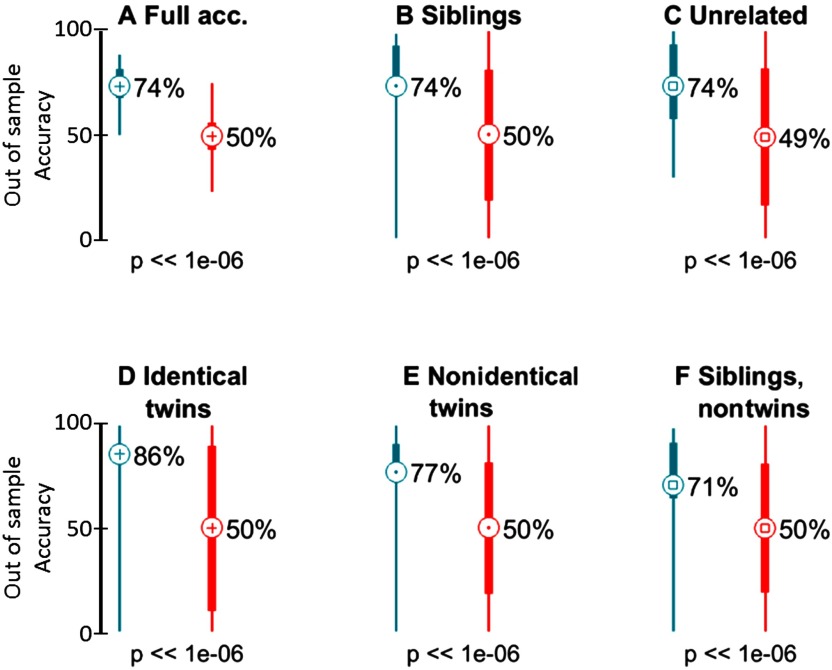
Familial patterns of brain connectivity captured in youth can also be seen in an independent sample of adults when using connectotyping. The figure depicts the accuracy (*N* = 1,000) of the connectotyping SVM classifier (blue) relative to permutation tests (red). (A) Full, (B) sibling, (C) unrelated, (D) monozygotic, (E) dizygotic, and (F) nontwin sibling accuracy is shown.

As a final validation of our approach, we trained classifiers in one dataset and predicted siblings and unrelated pairs in the other. As each dataset comes from different institutions, they have large differences in terms of imaging protocols, length of acquired data, processing and denoising strategies, and age of the participants (youths versus adults). Therefore, accurate predictions would signify the detection of shared environmental and genetic effects that are strongly generalizable. Per training dataset, all the sibling pairs and an equal number of unrelated pairs (resampling on each run) were included. Classifiers were used to predict all the sibling pairs of the other dataset and an equal number of randomly selected unrelated pairs. Interestingly, only youths predicting adults by using connectotyping rendered significant results (see Figure 5 and Figure 5-Figure Supplement 1, Miranda-Dominguez et al., 2018). In this case, the average out-of-sample accuracy was 74%, with a sensitivity of 74% and a specificity of 74%, *p* < 10^−6^ for all. Interestingly, and also in line with heritability, the classifiers’ accuracy was higher for identical twins (86%) than for nonidentical twins (77%; *p* < 10^−6^ rank-sum test, Cohen’s *d* = 0.27) and nontwin siblings (71%; *p* < 10^−6^ for all as shown in Figure 5D–F). These results obtained using an independent dataset again suggest heritable effects that constrain FC patterns.

### Classification of Sibling Pairs Using Anatomical Features

To be sure that the above findings were not driven by underlying anatomical distinctions instead of patterns of functional connectivity, we repeated the previous analysis (i.e., we trained classifiers in youth and predicted siblings and unrelated participants in adults) using cortical thickness and sulcal depth, independently. We first trained the anatomical classifiers using the same ROIs (i.e., features) utilized for the functional analysis above. If our results were driven by anatomical features instead of functional relationships, repeating our analysis using anatomical, instead of functional, measurements for the same ROIs should lead to a successful classification between siblings and unrelated participants. Instead, we found that neither sulcal depth nor cortical thickness was able to distinguish between siblings and unrelated pairs. This was true for the raw anatomy data and for data corrected by intracranial volume (using normalization or regression, separately).

**Figure 6.**
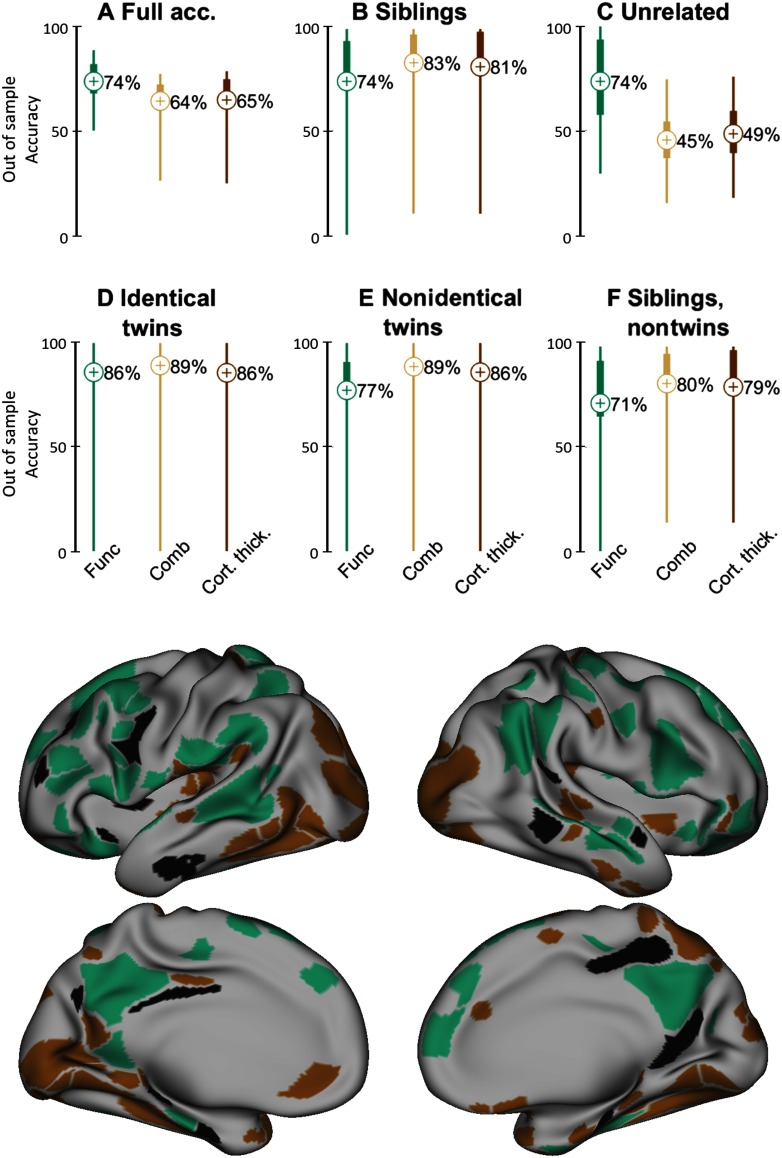
Comparing the performance of classifiers using connectotyping (green) and anatomical features cortical thickness (brown) to classify kinship in adults when classifiers were trained using data from an independent dataset of youths. Green traces correspond to the results of the classification using connectotyping, as shown in Figure 5. The same classification procedure was repeated using cortical thickness as features (after removing the effect of head size), but using the top 100 more distinct features according to connectotyping (light brown, labeled as “Comb” to indicate “combined”). Dark brown lines show the performance of the classifiers when features and feature selection were based on cortical thickness. Bottom panel (brain figure): Top most distinct features between siblings and unrelated participants (youth) for connectotyping are shown in green. Brown ROIs are the top distinct features according to cortical thickness. ROIs in black (*N* = 20) correspond to the overlapped regions between connectotyping and cortical thickness.

Next, we reran the analyses from scratch. This time we used the ROIs or features for the anatomical analysis that most separated the samples in the training set on each run. We found that while small levels of out-of-sample accuracy could be identified, the results were lower compared with connectotyping, and the classifiers tended to overpredict siblings at the cost of unrelated pairs. Not only that, the identified features showed little overlap with the functional features, suggesting that while anatomy might be familial, such traits may be distinct from the functional familiality. Figure 6 shows the case with the best out-of-sample performance: cortical thickness, normalizing anatomical features by head size. The other conditions, which included no normalization, or regressing out head size, showed worse classification (Figure 6, Figure Supplement 1, Miranda-Dominguez et al., 2018).

### Quantifying the Heritability of the Human Connectome

**Figure 7.**
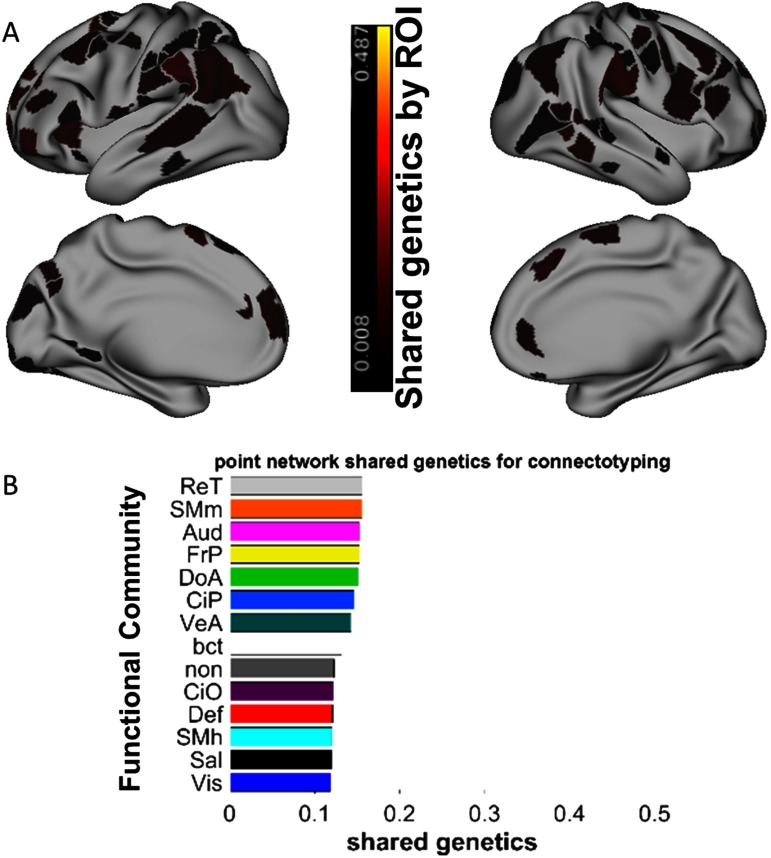
Heritability of connectotyping-based functional connectivity. (A) The connectotyping heritability of the top 100 regions used in the SVM classification for connectotyping (see Figure 3). (B) The connectotyping heritability for each network. Networks are sorted from most to least heritable, and the bar color matches the networks shown in Figure 2.

Heritability was then quantified for connectotyping measures using three-way (shared environment × shared genetics × ROI) repeated-measures ANOVA (analysis of variance), with ROI as the repeated measure. Heritability estimates (shared genetics) were made at the level of each of the 352 individual regions (333 cortical + 19 subcortical), each of the 14 networks, the whole brain, and for all individual ROI-ROI correlations (Figure 7-Figure Supplement 1, Miranda-Dominguez et al., 2018). We found that connectotyping predictions showed significant but small heritability for 257 of the 352 individual ROIs (*h*^2^ < 0.05, *p* < 0.05 corrected for multiple comparisons). Figure 7 plots the heritability of the top 100 features of the SVM for connectotyping. Notice that heritability is low at the level of individual regions.

Therefore, we examined the heritability across the whole brain and for each network controlling for the effect of individual ROIs via repeated-measures ANOVA. We found that the dorsal attention and fronto-parietal systems were among the most heritable (Figure 7A, top; *h*^2^ > 0.14, *p* < 1*e* − 6), paralleling the most used networks for SVM. Thus, the SVM is likely capturing some heritability of individual networks. Across the whole brain, heritability was much greater (*h*^2^ = 0.20 (upper 95% CI = 0.25, lower 95% CI = 0.14; *p* < 1*e* − 6) than at the level of individual regions or networks and further suggests that rather than individual connections, groups of functional connections are heritable.

Using the same repeated-measures ANOVA, we found that the shared environment of networks was greater for the whole brain than for individual connections (Figure 7-Figure Supplement 1, Miranda-Dominguez et al., 2018).

Connectotyping predicting time courses show significant heritability and shared environment in both the 198 (heritability: Figure 7; shared environment: Figure 7-Figure Supplement 1, red plus sign, Miranda-Dominguez et al., 2018) and 499 (heritability: Figure 7-Figure Supplement 1, blue circle; shared environment: Figure 7-Figure Supplement 1, blue plus sign, Miranda-Dominguez et al., 2018) datasets. Taken together, the set of results suggests that connections between groups but not pairs of brain regions may be heritable. However, these results are difficult to interpret because nontwin sibling pairs represent a large portion of our data, so we are reluctant to interpret these results with too much emphasis. A twin design with greater numbers than presented here would be required to properly estimate shared environment.

## DISCUSSION

### Individual Connectomes Can Be Reliably Identified Across Many Years, Even in Youth

In a previous study (Miranda-Dominguez, Mills, Carpenter, et al., 2014) we showed that even 2.5 min of fMRI data can be used to build robust, individual connectotypes in adult humans and in nonhuman primates. Those individual signatures were able to identify individuals with very high reliability after a 2-week interval, mainly because of activity in brain systems involving fronto-parietal areas.

In the current report we extended these findings in several ways. First, we showed that connectotypes can uniquely identify children, even when scanned over 2 years apart, which suggests that individualized FC patterns are preserved and superimposed on top of developmental changes in brain connectivity. Second, we distinguished individual sibling and twin pairs from unrelated pairs in both children and adults, demonstrating that family status constrains the functional organization of an individual’s brain. Consistent with our prior findings, familial status was most prominently classified by higher order systems, including the fronto-parietal, dorsal attention, and default systems. Last, after quantifying heritability, we showed that rather than individual connections, clusters of connections show modest heritability.

### Connectomes Are Familial and Heritable

The present work represents a fundamentally novel approach to testing familial (influences on the connectome due to shared genetics and/or shared environment heritability) and heritable (influence on the connectome implied by shared genetics) aspects of functional brain networks. Heritability studies often rely on forms of regression, such as structural equation models (Fu et al., 2015) or general linear mixed models (Visscher & Goddard, 2015). Because brain regions are interconnected, and many brain regions exist within the brain, approaches that examine heritability of single connections or brain regions may not capture network-level effects appropriately. Here, we tested familiality using SVMs and heritability using traditional ANOVAs. As can be seen by our heritability analysis, most individual connections show limited variation in heritability, which is consistent with the prior literature (Fornito et al., 2011; Glahn et al., 2010; Korgaonkar et al., 2014). In contrast, individual networks and whole-brain networks were far more heritable. These analyses represent only a subset of possible multivariate approaches, and therefore it should be interesting for future work to evaluate alternative multivariate models (e.g., latent factor analysis) that may be amenable to more traditional heritability analysis.

Our findings are consistent with prior rs-fMRI studies that have found significant heritability for whole-brain network or organizational properties (Sinclair et al., 2015; van den Heuvel et al., 2013). Yang et al. (2016) and Fu et al. (2015) found significant within-network heritability for most brain networks, but heritability was limited between functional networks (Yang et al., 2016). Heritability of correlations within networks in the present study were comparable to these prior studies. The SVM model used here, however, can also be validated in different datasets, which overcomes a strong criticism of most heritability studies. Here we found that connectotyping-trained SVMs, but not correlation-trained SVMs, could predict family status in completely independent datasets. Such an approach is in its infancy, so statistical geneticists will benefit from studying this approach in other contexts as well (e.g., behavioral studies or examining networks of genes instead of brain networks).

The present data demonstrate shared family characteristics of the connectome in two independent datasets with large differences in age as well as in acquisition and processing parameters. Interestingly, while the child and adult groups exhibited slight differences in the relative contributions of different brain networks toward sibling classification, the top network contributors were similar (see below). This finding suggests that the familiality of the connectome is mostly determined by the same functional networks, regardless of age.

Importantly, machine learning could offer an alternative approach to estimate heritability. Training classifiers to distinguish between mono- and dizygotic twins could disambiguate between the contributions of shared environment or shared genetics in the estimation of heritability of multiple traits.

### Familial Relationships Are Driven by Shared Variance in Higher Order Systems

We found that the brain networks that contributed most toward familial classification included the default mode, cingulo-opercular, ventral attention, dorsal attention, and fronto-parietal systems. The contribution of the fronto-parietal and dorsal attention networks to familiality was larger in the adults than in the youth. This finding sheds light on our prior work that showed that the fronto-parietal and dorsal attention systems have the most variable FC patterns in adults (Miranda-Dominguez, Mills, Carpenter, et al., 2014), a finding replicated in multiple contexts (Finn et al., 2015; Mueller et al., 2013). While the discrepancies between children and adults must also be taken in light of differences in data acquisition, the increased contribution of these fronto-parietal systems might represent changes in gene expression and topological reorganization of the brain across age. For example, recent work has shown that the expression of genes involved in synaptic function correlates with the strength of FC across the lifespan (Richiardi et al., 2015), potentially contributing to the reshaping of systems.

The cingulo-opercular and ventral attention systems (along with the fronto-parietal networks noted above) have long been known to be involved in higher order executive or attentional phenomena (Corbetta & Shulman, 2002; Dosenbach et al., 2007; Power, Fair, Schlaggar, & Petersen, 2010). The ventral attention and dorsal attention networks work together in the dynamic control of attention in relation to top-down goals and bottom-up sensory stimulation (Shulman, Ollinger, Linenweber, Petersen, & Corbetta, 2001). The cingulo-opercular system is also involved in the maintenance of task sets (Dosenbach, Fair, Cohen, Schlaggar, & Petersen, 2008), among other higher order cognitive phenomena such as control initiation and adjustment in response to feedback (Dosenbach et al., 2006, 2008). Indeed, the clusters of regions identified here as being heritable or familial are largely overlapping with brain regions that covary with multiple features related to psychometrics, behavioral measures, and demographics—relationships that relate to general intelligence and “real-life” function (such as cognition, life satisfaction, years of education, income; Smith et al., 2015). The observation that complex patterns of activity within and between these networks are uniquely heritable may help explain why complex behaviors associated with these systems are also highly heritable (Friedman et al., 2008).

An important consideration is that these results do not seem to be dominated by the size of the functional networks per se. Figure 3-Figure Supplement 1 (Miranda-Dominguez et al., 2018) shows that even small functional networks have a strong contribution to the familiality and heritability of the connectome. Furthermore, when classifiers were run using only ROIs from each functional network independently, large sensorimotor systems like the visual, auditory, and somatosensory hand ranked low on their ability to classify siblings from unrelated participants (Figure 3-Figure Supplement 4, panel B; Miranda-Dominguez et al., 2018). We also did not find evidence that our approach could be biased by ROI size since there were no differences in terms of ROI sizes when comparing the ROIs included or excluded (Kolmogorov-Smirnov test, *k* = 0.1169, *p* = 0.3041, Figure 3-Figure Supplement 3, Miranda-Dominguez et al., 2018) in the classifiers.

### Age Differences in Familiality and Heritability

This study benefited from the training and testing of a machine-learning model using independent datasets. The finding that heritability could be identified in adults after training in youth provides two important insights. First, since the youth data consisted of much shorter scan times, this supports the notion that connectotyping extracts information from limited datasets that may not be captured by traditional functional connectivity correlation matrices (Miranda-Dominguez, Mills, Carpenter, et al., 2014). Second, classifying siblings could not be done when training the SVM on adults and predicting siblings in youth. Multiple factors may have contributed to differences in performance of our classifiers, including site and data acquisition procedures (see limitations below). Nonetheless, the ability to classify sibling status in adults from models generated in children, but not children from models generated in adults, is intriguing and deserves further study. One possibility of this lack of generalization might be due to the specific features chosen for the training data—whether they come from children or adults. Fronto-parietal systems, which formed the core of models in many respects, are important for functional fingerprinting (Miranda-Dominguez, Mills, Carpenter, et al., 2014), and diverge in individuals over the course of development into adulthood. In short, fronto-parietal systems take longer to fully mature (Gogtay et al., 2004). If so, the top selected features for training from the child set might constitute a distinct subset of that produced by the adults. Presumably, while not ideal this subset could still be used to predict sibling status because they are also part of the “functional fingerprint” in adults. On the other hand, the features that best estimate sibling relationships from adults might include some features that are already developed in children, but others that are not yet fully developed as well. In such a scenario, the mature pattern (or feature set) would not be optimal, and indeed might fail in children retrospectively for classification. Work across sites within adult samples, or within child samples, will assist in testing these ideas.

### Functional Fingerprinting

The current work builds on new trends in neuroscience aimed at identifying individual signatures of complex brain connectivity. The current framework is distinguished from other conventional FC approaches in a number of salient ways (Friston, 2011). First, traditional measures of pairwise association such as Pearson correlations are confounded by correlations with other brain regions. While this cofound can be minimized by using partial correlations, FC is a measure of statistical dependencies among ROIs. Via multiple linear regression, connectotyping attempts to determine the unique contributions made between each pair of brain regions; in other words, connectotyping attempts to explain the statistical dependencies observed in FC. Second, connectotyping provides an equation to predict brain signal in future data. Third, recent literature suggests that at least 30 min of motion-free resting-state data are required to obtain a functional correlation matrix that converges closely with its final form averaged over many scanning sessions (Laumann et al., 2015). This is far greater than the amount of data typically acquired, and it may not be a realistic goal for many studies. It is likely that the variability of correlations with limited data contributes substantially to the difficulty in obtaining reproducible results in studies of complex neuropsychiatric illnesses (Castellanos, Kelly, & Milham, 2009), particularly in studies of neurodevelopment because of the greater incidence of head motion in younger participants (Satterthwaite et al., 2012; Van Dijk, Sabuncu, & Buckner, 2012). Our results demonstrate that connectotyping models calculated with as little as 2.5 min of data (Miranda-Dominguez, Mills, Carpenter, et al., 2014) enable sophisticated predictions that can be validated in independent datasets. This may increase the usability of new as well as currently available resting-state datasets.

When comparing by visual inspection traditional connectivity maps obtained by correlations with beta weights obtained by connectotyping (Figure 1-Figure Supplement 1, Miranda-Dominguez et al., 2018), we advise against judging the beta weights based on how well they can differentiate functional networks. Functional networks are defined following data-driven approaches that take as input traditional correlations among brain regions. Hence, correlations represent the optimal solution to visualize such networks. Connectotyping is tuned to capture the global linear dependence across ROIs. This means that each coefficient needs to be considered together with all the other beta weights. It is also important to mention that these beta weights are the result of a regularized but underdetermined multiple regression (in other words, the number of ROIs outnumbers the number of frames, i.e., the degrees of freedom per time course). This fact means that no optimal connectotyping solution for a given individual might be found when acquisition times are short. However, as shown here and in the original connectotyping manuscript (Miranda-Dominguez, Mills, Carpenter, et al., 2014), the estimated connectotype strongly represents individualized signatures, and the areas that vary the most (and the least) have a biological interpretation.

There are other substantial implications to the connectotyping approach. The connectotype model exposes latent similarities (and dissimilarities) between individuals due to kinship (or due to lack of kinship). In principle, these individualized signatures may be useful in identifying atypical patterns of brain functioning in ways that are heterogeneous or obscured by traditional group-level analyses (Fair, Bathula, Nikolas, & Nigg, 2012). Thus, the approach outlined here provides new avenues for a critical goal of modern neuropsychiatric research: to elucidate how unique patterns of neuropathology may manifest as related clinical outcomes (Fair, Nigg, et al., 2012).

### Functional Fingerprints Are Determined by Patterns of Functional Connectivity, Not by Anatomy

To account for variation in anatomy, we used state-of-the-art surface-based registration pipelines (Glasser et al., 2013). Furthermore, when we repeated our analysis using anatomical features instead, the classifiers using anatomical features did not generalize and the selected features had little overlap (∼20%) with the functional features. Putting all these findings together, although additional work will need to be conducted in this realm, we can reliably demonstrate that our findings are not driven by pure anatomy. With that said, any study of functional connectivity using group-based derived parcellation schemata can potentially be affected by interindividual differences in area location. The delineation of individualized functional areas is probably the most effective approach to account for this potential confound. Definition of such individualized areas is still under investigations; however, as of now, any true correspondence likely requires at least 1 hr of high-quality functional data (Glasser et al., 2016; Laumann et al., 2015), something that is not feasible in most of MRI studies, particularly in special populations.

### Limitations and Considerations

While this study covers an important developmental time span (7–14 years), further research will be required to determine how early in development (prenatal/postnatal) connectomes acquire individualized characteristics (Graham et al., 2015). Future work will also be needed to determine whether effects are robust beyond a 2-year interval between scan sessions.

We acknowledge that consensus remains elusive regarding the optimal processes for acquiring and analyzing fMRI data, and more work is required to determine optimal denoising procedures (Burgess et al., 2016; Glasser et al., 2013). We used different methods on Oregon and HCP datasets to attenuate motion artifact, although both are recommended practices. Motion censoring combined with global signal regression was used for the Oregon dataset, while the Human Connectome Workflow Pipelines and strict quality control measures were applied to the HCP data along with global signal regression (Burgess et al., 2016; Siegel et al., 2016). Although this substantially limited our HCP sample size and introduced differences in processing with Oregon, the intent was to employ the processing strategies that were optimal for each institution’s set of acquisition parameters. We are also keenly aware of the need for imaging studies to be reliable and reproducible (Collins & Tabak, 2014). The use of publicly available datasets (in our case the HCP) to validate findings from our institutional data helped with this goal. In addition, our multivariate modeling procedure predicts “fresh, unseen” data, which represents a step toward maximizing reliability and reproducibility of findings. Finally, a recent rs-fMRI study showed that head motion may be heritable (Couvy-Duchesne et al., 2016). However, we did not find evidence of heritability of movement parameters (see Supplementary Information, Miranda-Dominguez et al., 2018).

It is also worth noting that between-subject differences in FC may be modulated by differences in functional architecture. Future developments in obtaining individualized brain parcellations from rs-fMRI data (Glasser et al., 2016; Laumann et al., 2015) will be critical for clarifying these distinctions, although such approaches currently require acquisition times that are prohibitively long for large-scale studies. Results reported here used the Gordon parcellation schema (Gordon et al., 2014). This ROI set outperforms many other well-established parcellations with varying numbers of ROIs in terms of homogeneity when defining functional areas (Gordon et al., 2014), indicating its robustness to variable functional architecture. Furthermore, our Supplementary Information, which compares the performance of connectotyping using different parcellation schemes (see Figure 1-Figure Supplement 3, Miranda-Dominguez et al., 2018), demonstrates that the predictability of siblings, same individuals, and unrelated pairs is largely independent from the ROI set. Taken together, such findings make it unlikely that the SVM model is driven by how well each participants’ ROIs were mapped into the anatomical atlas.

An important consideration in this study is that the two datasets used for cross-validation differ in multiple dimensions: scanner hardware, parameters of the imaging sequences, pre- and postprocessing methods to account for noise, as well as the length of the resting-state time courses. The usage of more homogeneous datasets might lead to higher out-of-sample accuracies than the ones we found here. On the other hand, the fact that the two datasets differ so substantially extends the credibility that our findings are generalizable.

One factor that might have been particularly affected by the two independent datasets for our analyses was the seemingly discrepant finding that the model-based approach was stronger at characterizing family relationships (as opposed to traditional correlations); on the contrary, the quantification of heritability was stronger with traditional correlations. There are a few considerations that might explain these findings. Our analysis using machine learning includes both datasets, while the formal estimation of heritability was done using only the sample of adults. Under this context it is likely that the discrepancy between the results is largely a function of the differences in the acquisition parameters, in particular, the amount of data collected. Connectivity in the youth dataset was characterized using 2.5 min of movement-free resting-state data, while in the adult datasets 1 hr of data was available. Hence, traditional correlations in the adult dataset are likely a good representation of individual connectivity (Laumann et al., 2015). As mentioned before, connectotyping coefficients are the result of a regularized but underdetermined multiple regression leading to more variable correlations between predicted and observed time courses. Potentially, because the higher normalized variance decreases effect size estimates for the ANOVA in the heritability analysis, the heritability estimates are lower for the connectotyping-based predictions, as opposed to the traditional correlations. More work is needed on this front, but these data might suggest that when quantifying heritability of the connectome using traditional methods, if data of up to 1 hr are available, traditional correlations might be more suited.

Nonetheless, in traditional acquisition settings, both our current and our prior work suggest that the use of mathematical modeling and regularization tools can be used to effectively examine regional interactions from short scan sessions (a few minutes of data), enabling the reliable characterization of individuals. However, we recognize that other methods toward characterizing individual connectomes have been conducted (e.g., Shehzad et al., 2009). For example, it has been proposed that classifiers can be tuned to find group differences in functional MRI by modeling the nonlinear relationship between the BOLD signal and the neural activity. This approach is designed to map such differences to “hidden” physiological variables like synaptic weights (Brodersen et al., 2011). In addition, we recognize that our approach may not be optimal for all scientific inquiries and types of data. Indeed, short scan sessions (or data availability) are unlikely to be sufficient for identifying some detailed distinct characteristics of the individual connectome (Laumann et al., 2015; Poldrack et al., 2015).

### Conclusions

In this study we show that FC can be reliably used to identify and track individual brain signatures—a so-called functional fingerprint or connectotype for the human brain (Miranda-Dominguez, Mills, Carpenter, et al., 2014)—in both children and adults. These signatures demonstrate robustly familial and heritable relationships. Across development, these relationships are driven by specific higher order functional networks. Importantly, these signatures can be detected using limited data, are reliable, and are reproducible. Understanding the link between individual and familial connectomes may help us better describe the nature of heterogeneity across typical and atypical populations (Costa Dias et al., 2015; Fair, Bathula, et al., 2012; Gates, Molenaar, Iyer, Nigg, & Fair, 2014) and assist in characterizing the interactions of genetic and epigenetic factors toward brain function.

## METHODS

### Participants

Participants (*N* = 357) in this study were taken from two datasets: a youth longitudinal study in Oregon (*N* = 159) and the Human Connectome Project (HCP, *N* = 198).

#### Oregon.

Participants in this study (*N* = 159; 7.5–14.6 years old, mean = 10.64 and *SD* = 1.537, 60% males), free of known medical or neurological conditions (self-reported) and characterized to rule out ADHD and autism, were enrolled as part of an ongoing longitudinal study run at Oregon Health and Science University (131 participants with 1 scan, 27 participants with 2 scans, and 1 participant with 3 scans, *n* = 188 total scans). Parents provided written informed consent and youth under age 16 provided written informed assent. This sample contained 16 pairs of siblings, some of whom had multiple scans (Supplementary Table S1, Miranda-Dominguez et al., 2018).

#### HCP.

We used data from the HCP consortium “500 Subjects” release. We included the 198 out of 499 participants that passed our quality assurance (QA) criteria of having at least 5 min of usable data after strict motion correction (as described below). These were adult participants, age 22–36 years old, mean = 28.4, *SD* = 3.50, 45% males, belonging to 129 different families (70 families with one descendant, 49 families with two, and 10 families with three descendants). With regard to siblings, the data came from 10 pairs of monozygotic twins, 11 pairs of dizygotic twins, and 58 pairs of nontwins. Seventy participants had no siblings (Supplementary Tables S4 and S5, Miranda-Dominguez et al., 2018).

### MRI Data Acquisition

#### Oregon.

Structural and functional data were acquired using a 3T Siemens Trio Tim equipped with a 12-channel head coil. Resting-state data were acquired in three 5 min sessions using BOLD contrast (3.75 × 3.75 × 3.8 mm voxels, TR = 2,500 ms, TE = 30 ms, no acceleration factor). Subjects were instructed to lie still and fixate on a crosshair at the center of their visual field.

#### HCP.

Structural and functional data were acquired in a 3T Siemens Skyra scanner using a 32-channel head coil. Resting-state BOLD data were acquired with 2 mm (isotropic) voxels, TR = 720 ms, TE = 33.1 ms, and a multiband acceleration factor of 8. Complete acquisition details for Oregon and HCP are available in the Supplementary Information (Miranda-Dominguez et al., 2018).

### MRI Data Preprocessing

#### Oregon.

Data were processed using the pipelines from the Human Connectome Project (Glasser et al., 2013); see details in the Supplementary Information (Miranda-Dominguez et al., 2018). Resulting time courses (surface registration for cortex and volume registration for subcortical gray matter) were detrended and further processed to remove the effect of movement regressors (Friston, Williams, Howard, Frackowiak, & Turner, 1996; Power et al., 2014; Power, Barnes, Snyder, Schlaggar, & Petersen, 2012) and whole brain, ventricle, and white matter signals by regression. Finally, a first-order Butterworth band pass filter was applied to the time courses to preserve frequencies between 0.009 and 0.080 Hz (Fox et al., 2005).

Motion censoring: Signal from volumes where the total relative movement in any direction (frame displacement, FD) in relation to the previous volume were greater than 0.2 mm were censored out, as well as all surviving segments of data lasting fewer than five contiguous volumes (Power et al., 2012). Participants were included if at least 2.5 min of usable data survived motion censoring (surviving frames statistics: average = 242, *SD* = 83.2; range: 60 to 344). The 2.5-min threshold is based on prior work showing that our connectotyping models are quite stable for this amount of data (Miranda-Dominguez, Mills, Carpenter, et al., 2014).

#### HCP.

For this analysis, we used the ICA-FIX denoised rs-fMRI time courses provided by the HCP (Smith et al., 2013); see details in Supplementary Information (Miranda-Dominguez et al., 2018). Furthermore, we applied whole-brain regression to minimize the effect of structured noise (Burgess et al., 2016). To be sure that movement was not a concern for the current analysis, we selected a subset of 198 participants having at least 5 min of usable data with an FD of at most 0.2 mm. For the selected participants, we used all the functional data available (i.e., we did not perform motion censoring).

### Parcellations and Functional Networks

Time courses were calculated as the average signal within the ROIs defined by different parcellation schemata: Markov (Markov et al., 2014), Yeo (Yeo et al., 2011), Power (Power et al., 2011), Gordon (Gordon et al., 2014), and HCP (Glasser et al., 2016). The Markov parcellation is an anatomical, macaque-derived parcellation schema that was projected onto a surface of the human brain (Miranda-Dominguez, Mills, Grayson et al., 2014) and included for comparison purposes with previous findings (Miranda-Dominguez, Mills, Carpenter, et al., 2014). Yeo, Power, HCP, and Gordon are data-driven parcellations. The majority of analyses are conducted with the Gordon atlas because of its superiority in predicting familial characteristics (Figure 1). This parcellation schema defines 333 ROIs clustered in 12 functional networks (47 ROIs have no assignment). The functional networks are auditory, cingulo-opercular, cingulo-parietal, default, dorsal attention, fronto-parietal, retrosplenial temporal, salience, somatosensory hand, somatosensory mouth, ventral attention, and visual. In addition to these functional networks, we added the subcortical segmentations derived from FreeSurfer to define a subcortical network. Figure 2 shows the location of each network projected onto a surface template of the human brain as well as the number of ROIs that comprise them.

### Estimation of Single-Subject Functional Organization Using Connectotyping

In order to capture each individual’s unique functional fingerprint, we used a model-based approach, termed “connectotyping,” that is derived from a mathematical modeling among ROIs and is based on previously established procedures described in Miranda-Dominguez, Mills, Carpenter, et al. (2014; see Supplementary Information, Miranda-Dominguez et al., 2018, for using correlation matrices to capture functional connectivity). Connectotyping explains how the brain activity of each ROI can be predicted by the weighted contribution of the activity of all the other ROIs. Such weights are optimized by regularization and cross-validation (Miranda-Dominguez, Mills, Carpenter, et al., 2014). Briefly, the first step consists of removal of autocorrelations from each ROI. To note, we did not remove autocorrelations on HCP data, since that dataset has already been cleaned by ICA (independent component analysis). Next, the activity of each ROI is modeled as the weighted contribution of the other ROIs. Overfitting is minimized by using regularization using truncated singular value decomposition. The optimal number of singular values to be kept was selected by cross-validation. The result is a directed ROI ×ROI connectotype matrix that indicates the weighted contribution of the remaining ROIs used to explain the time courses of each ROI:

$$\begin{array}{lll} \left[\begin{array}{l} \begin{array}{l} {\hat{r}}_{1,i}\\ {\hat{r}}_{2,i} \end{array}\\ \begin{array}{l} \vdots \\ {\hat{r}}_{M,i} \end{array} \end{array}\right] & = & \left[\begin{array}{ll} \begin{array}{ll} 0 & b_{1,2,i}\\ b_{2,1,i} & 0 \end{array} & \begin{array}{ll} \ldots & b_{1,M,i}\\ \ldots & b_{2,M,i} \end{array}\\ \begin{array}{ll} \vdots & \vdots \\ b_{M,1,i} & b_{M,2,i} \end{array} & \begin{array}{ll} \ddots & \vdots \\ \ldots & 0 \end{array} \end{array}\right]\left[\begin{array}{l} \begin{array}{l} r_{1,i}\\ r_{2,i} \end{array}\\ \begin{array}{l} \vdots \\ r_{M,i} \end{array} \end{array}\right]\;, \end{array}$$

where *M* is the number of ROIs, *r*_*i*_ is the vector of the residuals of the BOLD signal at the ROI *i* (after removing autocorrelations) ${\hat{r}}_{i}$ is the predicted value for each *r*_*i*_, and *b*_*i*,*j*_ are the regularized coefficients (beta weights) that constitute the connectotype. (Additional details are in Miranda-Dominguez, Mills, Carpenter, et al., 2014.) Software is available upon request.

### Machine Learning–Based Identification of Siblings

We used support vector machine (SVM) to classify sibling pairs and unrelated pairs. On each SVM experiment reported here, each dataset was randomly partitioned 1,000 times into “training (in-sample)” and “testing (out-of-sample)” subsets. The size of the fresh “testing” is specified for each in the Results section. Classifiers were optimized by leave-one-out cross-validation (loocv) using only the dataset “training.” Resulting optimized classifiers were tested in the fresh dataset “testing” to determine their accuracy. This out-of-sample performance is reported on each experiment (see Supplementary Information, Miranda-Dominguez et al., 2018, for details). Matlab code can be found at https://github.com/DCAN-Labs/MachineLearning_SVM (Miranda-Dominguez, 2016).

#### Classes.

Comparisons across each scan-pair were grouped according to kinship: “siblings” or “unrelated.” These groups were used as labels in the classifiers.

#### Features.

A connectotype (model) was calculated for each scan and used to predict time courses for each other scan. The correlation coefficients between the predicted and observed time courses for each ROI were used as features for each comparison.

#### Anatomy-derived features.

We calculated the average sulcal depth and cortical thickness for each of the 333 ROIs defined by Gordon et al. (2014). For subcortical features, we used the volume of the ROI. These numbers were used directly, or were controlled for brain size by either regression or normalization of brain size (results are reported for each case). When comparing paired data (i.e., siblings or unrelated), features were subtracted and the sign of the difference was kept.

#### Null hypothesis.

Null hypotheses were generated *N* = 1,000 times by randomizing class labels within the training partition for each run as described in the training section above.

#### Feature selection.

For each classifier, we followed two distinct approaches to select the features to retain:

•  By functional network: We optimized classifiers including all the features included on each of the 14 functional networks derived from the Gordon parcellation, and we did so independently for each network.
•  By significance (based on the Kolmogorov-Smirnov test): Regardless of network assignment, a subset of *p* features was selected to optimize each classifier. This subset was selected after ranking all the features (data from the participants assigned to the partition “prediction” were not used to rank features) based on how different they were (between class; sibling or unrelated), according to the Kolmogorov-Smirnov test. We decided to use this test because it makes no assumptions on the corresponding subjacent probability distribution functions of the features (Loudin & Miettinen, 2003).

### Heritability Analysis

Because the SVM approach can test but not quantify heritability, we performed a heritability analysis adapted from standard tests of heritability (Visscher & Goddard, 2015). We used the distributions of connectotyping predictions as the outcome variables in two separate analyses. The analyses were performed on the HCP dataset and not the Oregon dataset, which has no twins. We also conducted these analyses on both the 499 full HCP dataset (Figure 7-Figure Supplement 1, Miranda-Dominguez et al., 2018) as well as the 198 participants that met QA.

The heritability and shared environment (Figure 7 and Figure 7-Figure Supplement 1, Miranda-Dominguez et al., 2018) of the connectotyping models were tested using three-way (shared environment × shared genetics × ROI) repeated-measures ANOVAs, with ROI as the repeated measure. Only the main effects of shared genetics and environment were tested. All outcome variables were transformed into normally distributed variables using a rank-based transform (Glahn et al., 2010). Monozygotic, dizygotic, and sibling pairs represented one level in the shared-environment factor, whereas unrelated pairs represented the other level. Shared genetics had three levels: one for monozygotic pairs, one for dizygotic and sibling pairs, and a third for unrelated pairs. To estimate shared genetics and environment across the whole brain, ROI was included as a within-subject factor at 352 levels. To estimate heritability and shared environment per network, only ROIs within a given network were included as dependent variables. Heritability and shared environment (see Supplemental Information, Miranda-Dominguez et al., 2018) were quantified by measuring the ratio of the given factor’s sum of squares to the total sum of squares. Because we had far more unrelated pairs than sibling or twin pairs, a matching subset of unrelated pairs (*N* = 58) was randomly selected from the larger subset per ANOVA. Per analysis, we generated 95% confidence intervals for heritability and shared environment from 1,000 ANOVAs, and we randomly sampled the unrelated pairs per ANOVA.

## ACKNOWLEDGMENTS

We thank Marc Rudolph and Dr. Alice Graham for their helpful comments in reviewing this manuscript.

## AUTHOR CONTRIBUTIONS

Oscar Miranda-Dominguez: Conceptualization; Data curation; Formal analysis; Investigation; Methodology; Project administration; Software; Supervision; Validation; Writing – original draft; Writing – review & editing. Eric Feczko: Formal analysis; Validation; Writing – review & editing. David S. Grayson: Formal analysis; Validation; Writing – review & editing. Hasse Walum: Formal analysis; Validation. Joel T. Nigg: Funding acquisition; Investigation; Resources; Supervision; Writing – review & editing. Damien A. Fair: Conceptualization; Funding acquisition; Investigation; Methodology; Resources; Supervision; Writing – original draft; Writing – review & editing.

## TECHNICAL TERMS

Connectome: A map of connections of a given system. An example is the structural or functional connections among different brain areas.
Heteromodal: Highly interconnected brain areas located in fronto-parietal and temporal cortex involved in processing and integration of sensory inputs.
Phylogenetically: The relationship among heritable traits across time used to characterize evolutionary relationships among species.
Machine learning: A field of study that aims to extract hidden patterns or to make predictions based on existing observations of a given system.
Parcellation: Grouping of different and independent brain regions that share a similar profile based on functional or anatomical criteria.
Multivariate statistics: The usage of multiple features or metrics to characterize a system.
Support vector machine: A special case of machine learning that maximizes separation between labels or classes after transforming the features using a given kernel.
Sensitivity: Ratio of true positives after comparing the predicted versus the real outcome.
Specificity: Ratio of true negatives after comparing the predicted versus the real outcome.
