# Supplementary material for: Heritability of the human connectome: A connectotyping study
Source: Netw Neurosci. 2018 Jun 1;2(2):175–99. doi: 10.1162/netn_a_00029 (PMC6130446; doi:10.1162/netn_a_00029)
Supplement: Supplementary file 1 [file netn-02-175-s001.pdf]

Miranda-Domínguez, O., Feczko, E., Grayson, D. S., Walum, H., Nigg, J. T., & Fair, D. A. (2017). Supplemental Data Items for "Heritability of the human connectome: a connectotyping study." *Network Neuroscience, 2*(2), 175-199. https://doi.org/10.1162/netn_a_00029

Heritability of the human connectome: a connectotyping study

Supplemental data items

Supplemental Results

In this section we report the findings obtained after characterized brain connectivity using Pearson's correlation and performing the same experiments done in the main manuscript.

Comparing consistency within individuals, between siblings, and across unrelated pairs using traditional functional connectivity matrices.

To assess the traditional pearson correlation-based FC approach, we compared how similar the connectivity matrices were between groups (Same individual, predicting data in a different scan session; Individual predicting a sibling; individual predicting an unrelated participant). Here instead of using the model to "predict timecourses" we simply calculated the spatial correlation coefficient between matrices (see Supplemental Methods for details) ending up again with as many correlations as ROIs for each scan-pair being compared. These spatial correlations were then averaged providing 1 mean correlation value for each paired comparison. The distributions of mean average spatial correlations across all pairs are shown in Figure 1-Figure Supplement 4 panel c (*Note: we also compared spatial correlations for the beta weights or connectotypes* and present the corresponding distributions in Figure 1-Figure Supplement 4 panel b). Then, we used a t-test to compare distributions for siblings and unrelated participants. Spatial correlations for both connectotyping and traditional correlations showed that sibling pairs were significantly more similar than unrelated pairs ($p < 1.23\times10^{-6}$ for connectotyping (Figure 1 – Figure Supplement 2b) and $p < 8.96\times10^{-3}$ for correlations (Figure 1 – Figure Supplement 2c).

Classification of simply pairs in youth utilizing traditional function connectivity matrices.

For traditional functional connectivity correlations, the average out-of-sample accuracy was 69%. This finding was significantly higher than null, $p < 10^{-6}$). Sensitivity was 68% and a specificity was 69%. The classifications were mainly driven by ROIs belonging to the following functional networks: cingulo-opercular, dorsal attention, default mode, and ventral attention (Figure 3-Figure Supplement 5, panels D-E). As above, we did not observe significant changes in accuracy when a different number of features were used for classification (Figure 3-Figure Supplement 6 panel A).

Classification of sibling pairs in adults using Human Connectome Project data using traditional correlations

Classification by traditional functional connectivity correlations rendered significant out-of-sample classification accuracies, as shown in Figures 4-Figure Supplement 3 and Figure 4-Figure Supplement 4 (difference in accuracy for identical versus non-identical twins was also significant: $p < 10^{-6}$ rank-sum test, cohen's d = 4.07). Overall, these results suggest that kinship substantially contributes to individualized patterns of complex brain organization. Furthermore, greater accuracy for predicting monozygotic vs dizygotic twins strongly suggests that these patterns are partially heritable.

Classification of sibling pairs using independent datasets

Our validation approach using one dataset to predict siblings vs unrelated was repeated using correlations. As shown in Figure 5-Figure Supplement 2*,* the overall accuracy with traditional correlations was low (56%, $p < 10^{-6}$ rank-sum test, cohen's d = 2.07).

Quantifying the heritability of the human connectome

Heritability was also quantified for correlation measures using three-way (shared environment X shared genetics X ROI) repeated measures ANOVAs, with ROI as the repeated measure. Heritability estimates (shared genetics) were made at the level of each of the 352 individual regions (333 cortical + 19 subcortical), each of the 14 networks, the whole brain, and for all individual ROI-ROI correlations (Figure 7-Figure Supplement 3).

Correlations showed significant heritability for 283 of the 352 individual ROIs ($h^2 < 0.05$, $p < 0.05$ corrected for multiple comparisons). Figure 7-Figure Supplement 2 plots the heritability of the top 100 features of the SVM for correlations. Heritability is also low at the level of individual regions.

Therefore, we examined the heritability across the whole brain and for each network for traditional functional connectivity correlations, controlling for the effect of individual ROIs via repeated-measures ANOVA. For correlations (Figure 7-Figure Supplement 2A, top; $h^2 > 0.49$, $p < 1e\text{-}6$) dorsal attention and frontal parietal systems were among the most heritable, paralleling the most used networks for SVM. Thus, the SVM is likely capturing some heritability of individual networks. Across the whole brain, heritability was much greater ($h^2 = 0.53$ (upper 95% CI = 0.57, lower 95% CI = 0.48; $p < 1e\text{-}6$) than at the level of individual regions or networks and further suggests that rather than individual connections, groups of functional connections are heritable

Using the same repeated measures ANOVA we found that the shared environment of networks was greater for the whole brain than for individual connections (Figure 7-Figure Supplement 3). However, these results are difficult to interpret because no-twin sibling pairs represent a large

portion of our data, so we are reluctant to interpret these results with too much emphasis. A twin design with greater numbers than presented here would be required to properly estimate shared environment.

For traditional correlations, no individual connection showed statistically significant heritability after correction for multiple comparisons. Nevertheless, we quantified heritability and environment from the generalized linear mixed model for the top 100 features used in SVM, which showed little variation in heritability (Figure 7-Figure Supplement 3, black circle) or shared environment (Figure 7-Figure Supplement 3; black plus sign).

Spatial correlations show significant heritability and shared environment in both the 198 (heritability: Figure 7-Figure Supplement 2; shared environment: Figure 7-Figure Supplement 3, red plus sign) and 499 (heritability: Figure 7-Figure Supplement 3, blue circle; shared environment: Figure 7-Figure Supplement 3, blue plus sign) datasets. Taken together, the set of results suggests that connections between groups but not pairs of brain regions may be heritable.

Motion familiality:

Because a prior study reported that the degree of motion in resting-state data may be heritable, we used a general linear mixed model to evaluate familiality in the OHSU dataset. As above, the "fitlmematrix" function in matlab was used to construct and test the generalized linear mixed model. Because the OHSU dataset contained no twins, we could not dissociate shared environmental from shared genetic effects. Therefore, the familiality matrix represents the familiality between subjects; sibling pairs have a correlation of 1 and non-sibling pairs have a correlation of 0. The eigenvalues derived from the familiality matrix were used as the random factor, with sex and age as covariates. Point estimates of familiality were calculated by

measuring the ratio of the familiality component to the total variance. Pre-censored and post-censored OHSU datasets were tested for motion familiality.

Supplemental Methods

MRI Data Acquisition:

Oregon: Structural images were obtained using a T1-weighted MP-RAGE sequence (TR=2.3s, TE=3.58ms, flip angle = 10°, TI = 900ms, voxel size = 1mm^3, 160 sagittal slices). A T2-weighted sequences was also acquired (TR = 3.2s, TE = 497ms, voxel size = 1mm, slices = 160) as well as magnitude and phase field maps to correct for geometric distortions due to susceptibility artifact. Resting-state functional BOLD images were acquired using a gradient-echo, echo-planar sequence (TR = 2500 ms, TE = 30 ms; FOV = 240 mm; flip angle = 90°; 3.75x3.75x3.8 mm). Full brain coverage was obtained with 36 contiguous interleaved 3.8 mm axial slices acquired parallel to the plane transecting the anterior and posterior commissure.

HCP: We note that HCP data was acquired on a 3T Siemens Skyra optimized to achieve 100 mT/m gradient strength. All the data was corrected to account for the non-linearities associated with the high gradient and the displacement of the isocenter in this optimized system. For further details see the HCP 500 Subjects + MEG2 Data Release: Reference Manual (WU-Minn, 2014) and (Glasser et al., 2013).

Two separate T1-weighted images were acquired and averaged, with a TR=2400 ms, TE=2.14 ms, TI = 1000 ms, FA = 8°, and ES = 7.6 ms. Two T2-weighted images were acquired and averaged with a TR=3200 ms, TE=565 ms. T1-weighted and T2-weighted images were acquired with a voxel resolution of 0.7 mm (isotropic). Resting state BOLD data were acquired using a gradient echo echo planar imaging sequences with 2mm^3 voxels, TR=720ms, TE = 33.1ms, and a multiband acceleration factor of 8.

MRI Data Preprocessing:

Oregon: Data were processed using the pipelines from the Human Connectome Project (Glasser et al. 2013), which include the use of FSL (Smith et al. 2004; Jenkinson et al. 2012; Woolrich et al. 2009) and FreeSurfer tools (Dale et al. 1999; Desikan et al. 2006; Fischl & Dale 2000). Briefly, gradient distortion corrected T1-weighted and T2-weighted volumes were first aligned to the MNI's AC-PC axis and then non-linearly normalized to the MNI atlas. Later, the T1w and T2w volumes are re-registered using boundary based registration (Greve & Fischl 2009) to improve alignment. Then, the brain is segmented using recon-all from FreeSurfer. Segmentations are improved by using the enhanced white matter-pial surface contrast of the T2-weighted sequence. The BOLD data is corrected for field distortions (using FSL's TOPUP) and processed by doing a preliminary 6 degrees of freedom linear registration to the first frame. After this initial alignment, the average frame is calculated and used as final reference. Next, the BOLD data is registered to this final reference and to the T1-weighted volume, all in one single step, by concatenating all the individual registrations into a single registration.

Surface registration. The cortical ribbon defined by the structural T1-weighted and T2-weighted volumes is used to define a high resolution mesh which will be used for surface registration of the BOLD data. This cortical ribbon is also used to quantify the partial contribution of each voxel in the BOLD data in surface registration. Timecourses in the cortical mesh are calculated by obtaining the weighted average of the voxels neighboring each vertex within the grid, where the weights are given by the average number of voxels wholly or partially within the cortical ribbon. Voxels with high coefficient of variation, indicating difficulty with tissue assignment or containing large blood vessels, are excluded. Next, the resulting timecourses in this mesh are

downsampled into a standard space of anchor points (grayordinates), which were defined in the brain atlas and mapped uniquely to each participant's brain after smoothing them with a 2mm full-width-half-max Gaussian filter. Subcortical regions are treated and registered as volumes. Two thirds of the grayordinates are vertices located in the cortical ribbon while the remaining grayordinates are subcortical voxels.

Nuisance regression. Additional preprocessing consists of regressing out the grey matter, ventricle and white matter average signal, and the movement between frames from the six image alignment parameters x, y, z, θ_x , θ_y, and θ_z on the actual and the previous TR and their squares, which correspond to the Volterra series expansion of motion (Power et al. 2014; Friston et al. 1996; Power et al. 2012). The regression's coefficients (beta weights) are calculated solely based on frames with low movement, but regression is calculated considering all the frames to preserve temporal order in the data for filtering in the time domain. Next, timecourses are filtered using a first order Butterworth band pass filter to preserve frequencies between 0.009 and 0.080 *Hz*.

HCP: For this analysis, we used the ICA-FIX denoised rfMRI timecourses provided by the HCP. These timecourses were minimally processed first by the HCP as described below. Next, they applied Independent Component Analysis (ICA) to account for nuisance and covariates with a new FSL tool named FIX that automatically removes artifactual or "bad" components. Briefly, each voxel's timecourses from 25 HCP subjects were decomposed into 229 spatial components. Of these, on average 24 components were hand-classified as "good" and the remainder as "bad". Next, a classifier was trained to identify "good" and "bad" components. Once the classifier was optimized (by leave-one-subject-out cross validation), the resulting classifier was used to identify

the "bad" components from each participant. Such components were removed by regressing the "bad" components (timecourses) out from the timecourses on each grayordinate.

Machine learning based identification of siblings

Classifiers: For each run (out of N), SVM classifiers with a Gaussian (or Radial Basis Function, RBF) were optimized by leave-one-out cross validation (loocv), unless otherwise stated. The optimization is calculated using gradient descent to minimize the out of sample classification error by optimizing the parameters "Box Constraint" and a "scaling factor" ("Kernel Scale") in SVM. For SVM, "Box Constraint" controls the maximum penalty imposed on margin-violating observations, and helps in preventing overfitting (regularization) (Abu-Mostafa et al. 2012). This parameter is directly related to the number of support vectors used for classification. Increasing this number decreases the number of support vectors at the cost of training time. "Kernel Scale**"** is a scaling factor applied to the predictor variables.

Estimation of single-subject functional organization using traditional functional connectivity correlation matrix

Correlation matrices were calculated for each participant included in this study by calculating the Pearson correlation coefficient of the BOLD activity (after motion censoring) for any pair of ROIs used on each parcellation schema. The result was a ROI×ROI correlation matrix *per* participant that were used to characterize individual FC. To compare each scan pair, we defined the "spatial correlation" per ROI as the correlation between two subjects' FC vectors, where each vector is simply the set of correlations between that ROI and every other ROI.

Features for SVM using traditional correlations: To extract a comparable feature set using the pearson correlation matrices, we defined the "spatial correlation" per ROI as the correlation

between two subjects' FC vectors, where each vector is simply the set of correlations between that ROI and every other ROI.

Heritability Analysis:

Traditional heritability: Because our heritability approach differs from prior studies, it was important to estimate the heritability of individual functional connections using traditional approaches from the literature as well. Therefore, the heritability of traditional correlations was tested using generalized linear mixed models (Visscher and Goddard, 2015) via the "fitlmematrix" function in MATLAB, where each functional correlation was used as the predicted measure. For comparisons to the SVM model (see: Figure 2), mean ROI heritability was calculated for each ROI by taking the average heritability for functional correlations between the given ROI and all other ROIs. Mean network heritability was measured by computing the average of the mean ROI heritability for all ROIs within each network. All measures were transformed into normally distributed variables using a rank-based transform (Glahn et al., 2010). To aid in computation and reduce the dimensionality of the data, eigenvectors, derived via singular value decomposition, from the kinship and shared environment matrices were random factors. Per matrix, an eigenvector exists per subject and represents the shared genetics or environment between that subject and every other subject. The kinship matrix represents the genetic correlation between each pair of subjects; monozygotic twins have a genetic correlation of 1, other siblings have a correlation of 0.5, while unrelated pairs have no correlation; the kinship matrix is doubled prior to being used as a random factor. The shared environment matrix was generated as a binary matrix where a 1 indicated that the given pair of subjects lived in the same household and a 0 indicated that the given pair lived in

separate households. Sex and age were used as fixed effects in the analysis. Model parameters were estimated using restricted maximum likelihood estimation. To determine whether the genetic associations significantly explained variance in functional connections, another mixed model, called the shared environment model, was tested that excluded the kinship factor. A chi-squared test of the difference in log likelihood between the two types of models determined whether the contribution of the genetic component was statistically significant. We used the false discovery rate (FDR) to correct for multiple comparisons (Benjamini and Hochberg, 1995). Point estimates of heritability were calculated from the genetic plus shared environment model by measuring the ratio of the genetic component variance to the total variance (Visscher and Goddard, 2015).

Supplemental figures.

Figure 1- Figure Supplement 1. Visualizing functional networks using connectotyping and traditional correlations. Panel a) shows the mean connectotype across individuals; panel b) shows a connectoype from 1 individual; and panel c) shows the correlation matrix of that individual. The "*y"* axis correspond to each ROI, sorted per functional network. The number shown in the "*y*" axis corresponds to the network's index, as coded in the table at the side.

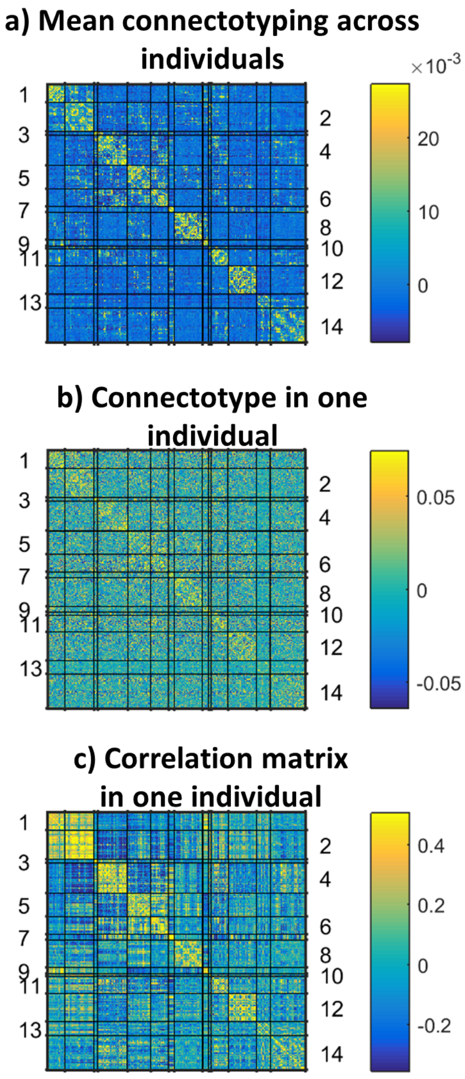

1) Auditory
2) Cingulo Opercular
3) Cingulo Parietal
4) Default
5) Dorsal Attention
6) Fronto-Parietal
7) Retrosplenial Temporal
8) Somato-sensory mouth
9) Somato-sensory hand
10) Salience
11)Ventral-Attention
12) Visual
13) Subcortical
14) No-assignment

Figure 1- Figure Supplement 2. Average correlation prediction when predicting timecourses from the same participant but months later. This figure corresponds to the group II from the figure I. This group is highlighted here to show in greater detail the low association between the average correlation coefficient of predicted timecourses and time between scans for connectotyping. This figure highlights the correlation coefficient between the average similarities between scans and time between scans following 3 criteria: 1) using all the data; 2) time between scans <=1.5 years; and 3) time between scans >1.5 years.

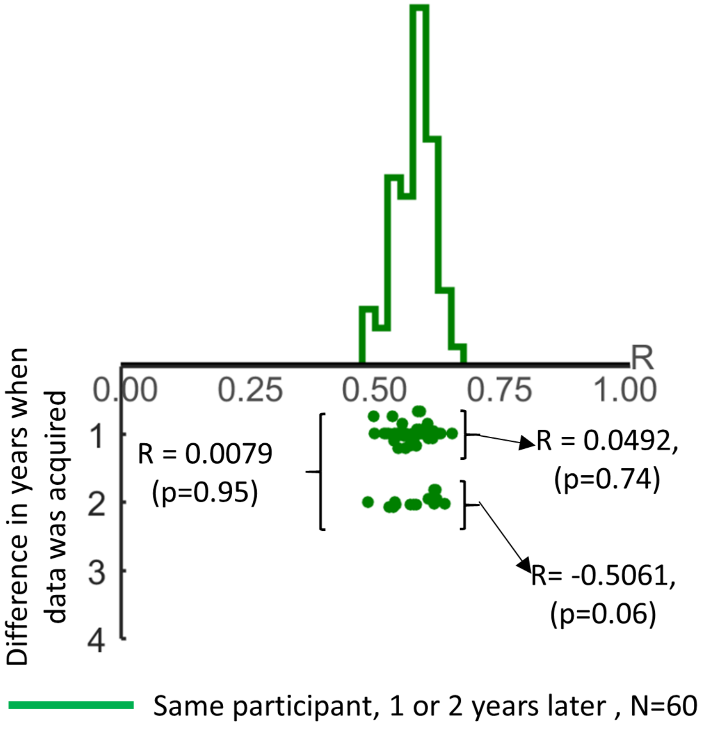

Figure 1-Figure Supplement 3. Segregating groups of paired data (same scan, same participant, siblings and unrelated) by predicting timecourses and spatial correlations. Panels "a" to "e" show the average correlation coefficient between predicted and observed timecourses across all participants under different parcellation schemas. Groups are based on the dataset used to calculate the model used in the prediction: I) the same scan session, II) the same participant but in a different scan session, III) a sibling, and IV) from unrelated individuals. Each panel also show the differences in age (measured in years) between each paired-data.

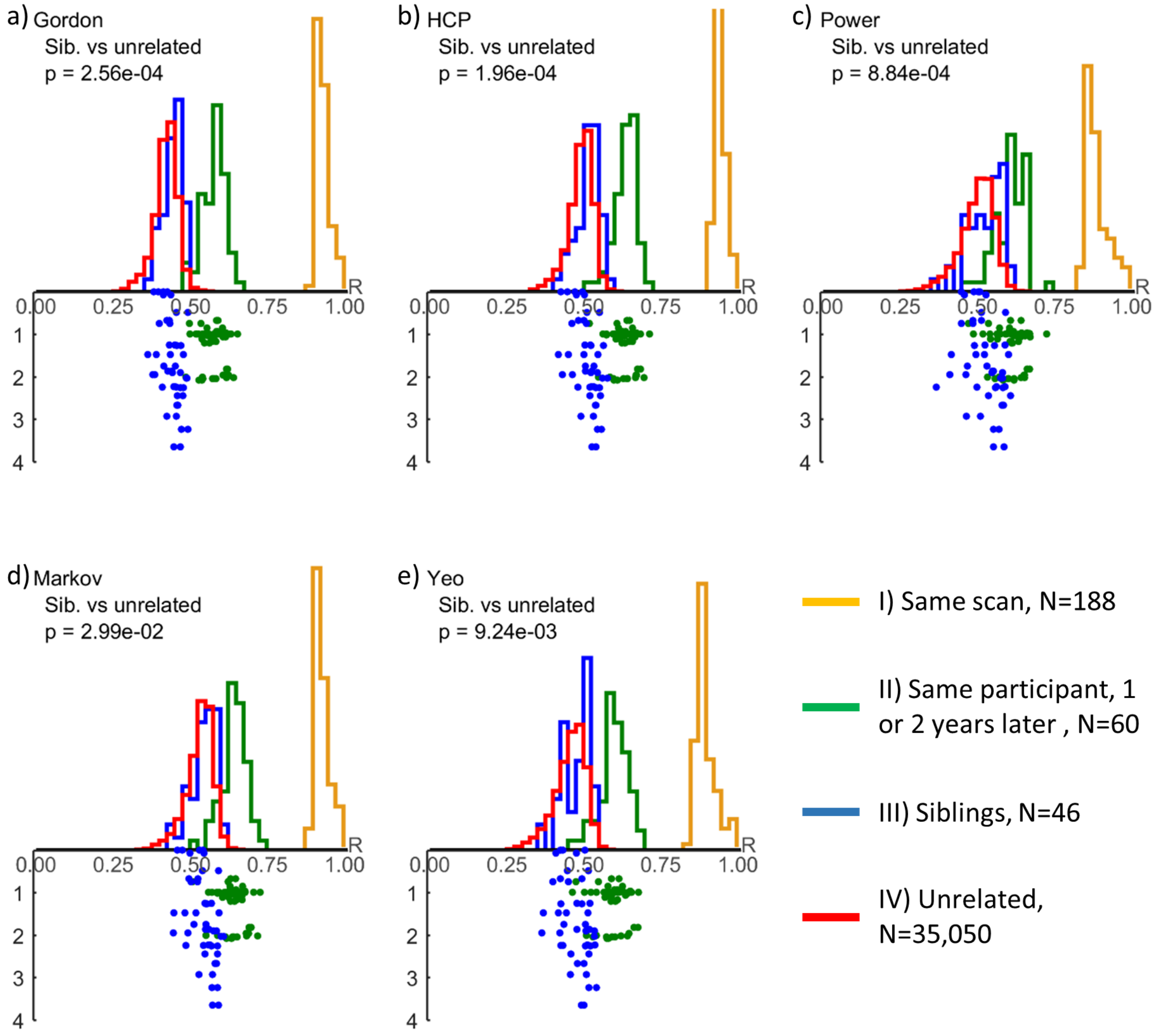

Figure 1-Figure Supplement 4. Spatial correlations. Panel a shows the results for connectotyping predicting timecourses and is repeated here for reference. Panels b and c show the spatial correlations using the Gordon parcellation for connectotyping (b) and traditional correlation matrices (c).

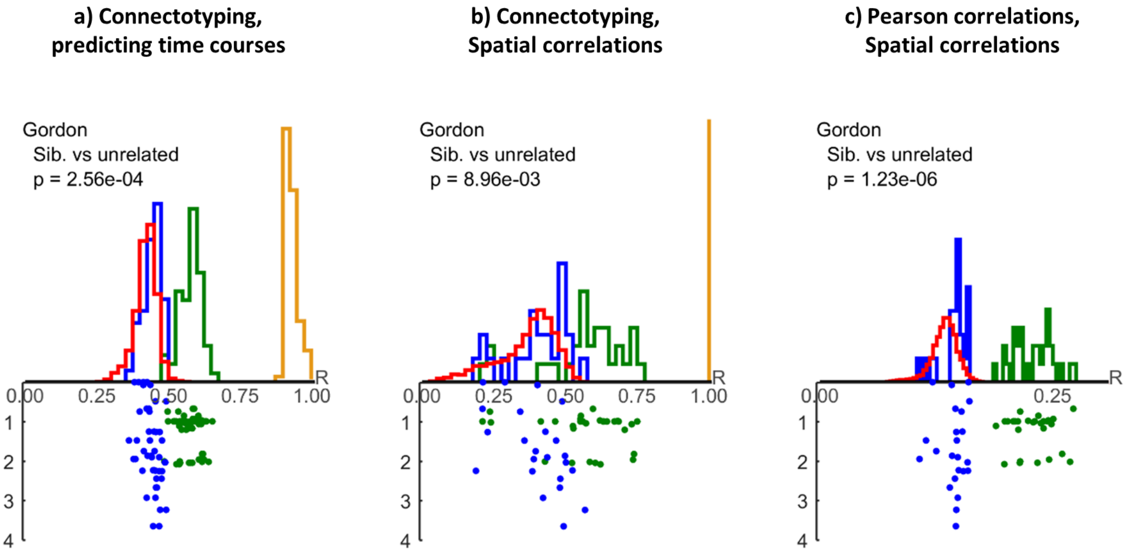

Figure 3-Figure Supplement 1. Top ROIs per functional network. Panel a) shows their location in a cartoon's brain. Panel b) shows the top features sorted per functional network (as in Figure 3). Panel c) shows the same top 100 ROIs sorted as the ratio of the number of ROIs identified in the network relative to the given network's size.

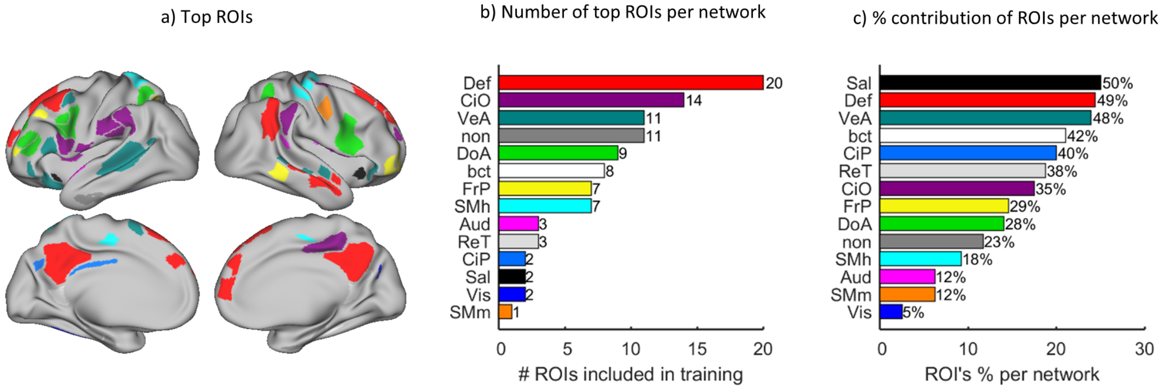

Figure 3-Figure Supplement 2. Mapping the 11 ROIs (within the top 100) with no proper assignment in Gordon into Yeo. Panel a shows the 11 ROIs within the top 100 with no functional assignment on top of the functional communities as defined by Yeo (Yeo et al., 2011) (also see tables S2 and S3). Colorcode shown in the bottom. Panel b shows the same information into a spherical projection of the brain.

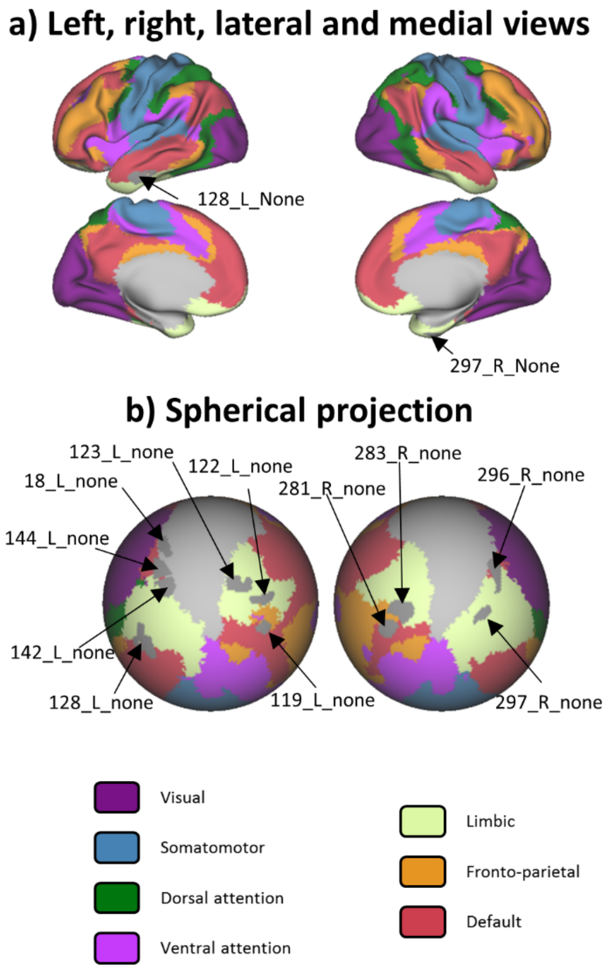

Figure 3-Figure Supplement 3. Distributions of ROI's size for the Gordon (+ subcortical) parcellation schema. Panel a) shows the distribution of the top 100 ROIs size (black) and the remaining 252 (red), as accounted by their number of grayordinates (see Table S2). Panel b) splits such distributions *per* functional network.

a)

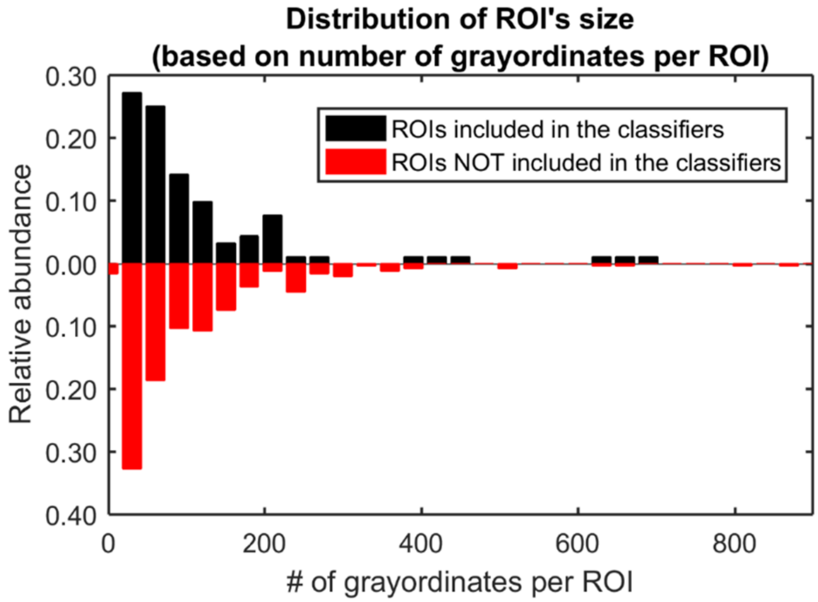

b)

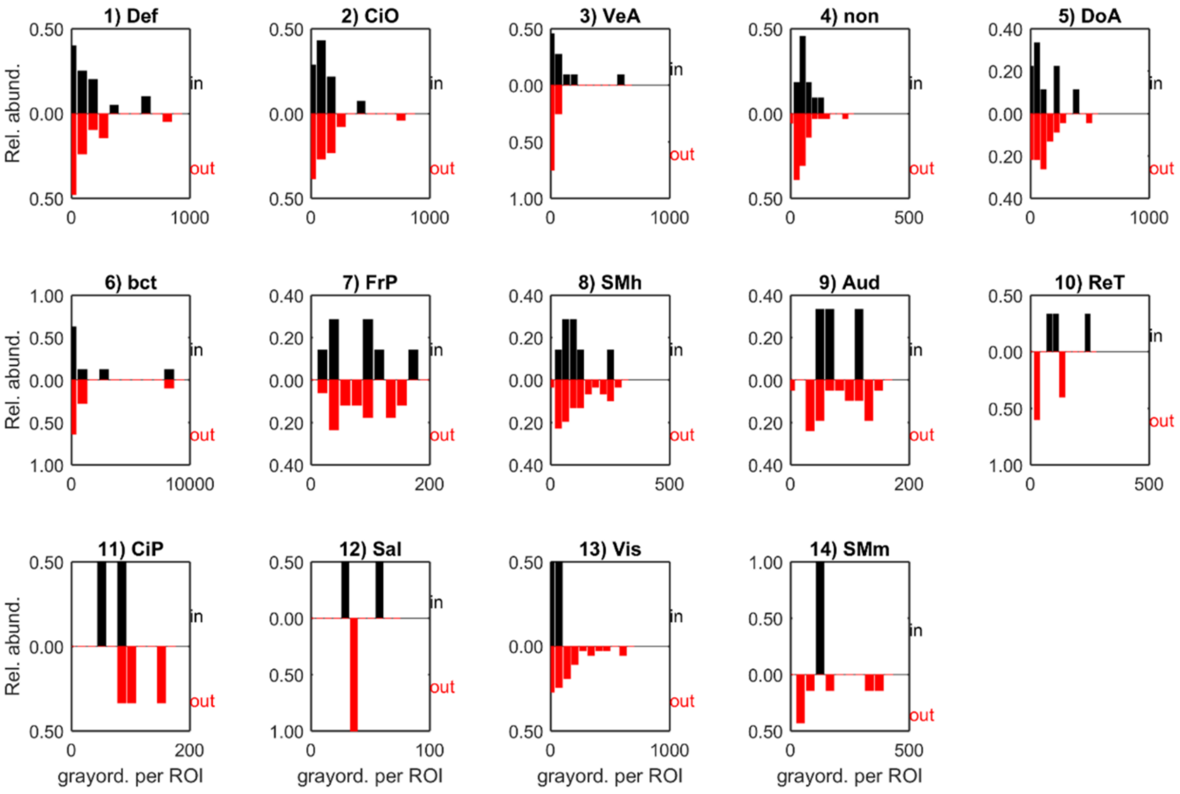

Figure 3-Figure Supplement 4. SVM's out of sample performance for CONNECTOTYPING and correlations, N=1000 per feature set. Mean full accuracy (+), mean accuracy predicting siblings (•), and mean accuracy predicting unrelated population (□). Panel A shows the out of sample performance of the SVM classifiers for connectotyping as a function of the number of features (x-axis) used for classification. Left-most panel shows the average results as a function of the number of features used for classification. Right-side subpanels show the distributions of accuracy, specificity (accuracy predicting siblings), and specificity (accuracy predicting unrelated participants) compared with the null hypothesis. Panel B show the corresponding results for connectotyping when the features were selected by functional network, as defined by Gordon (See Figure 2).

A. Connectotyping, per top features, out of sample accuracy.

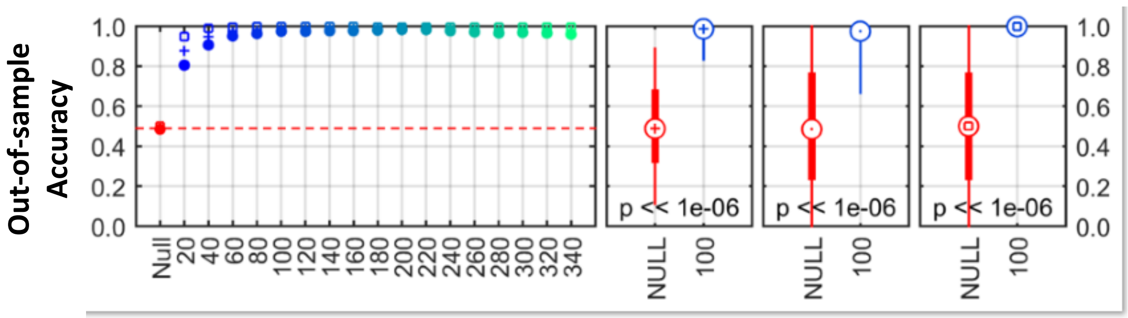

B Connectotyping, per network, out of sample accuracy.

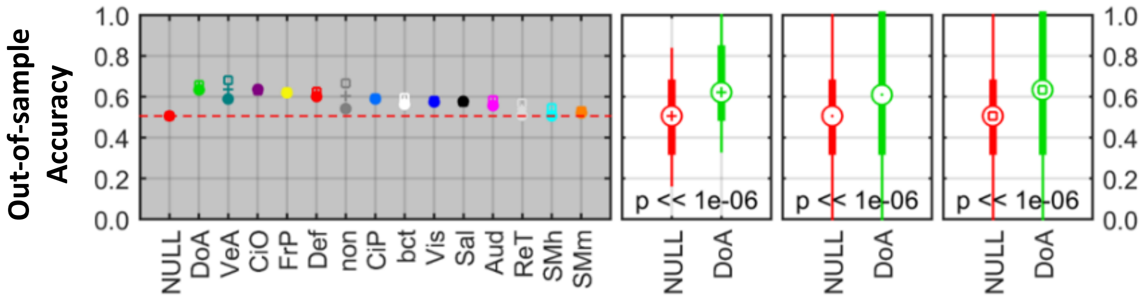

Figure 3 –Figure Supplement 5. Classifying siblings vs unrelated populations when other sibling pairs of the same family are included in the training set (sample of youths) characterizing brain connectivity using Pearson correlations. Distributions of (A) full accuracy, (B) siblings, and (C) unrelated pairs for the SVM when traditional correlations are used to characterize brain connectivity. The bottom left panel shows the consensus' ROI's per functional network (as defined by Gordon) used in the classifier, and the location of such ROIs in the surface of the brain. Each distribution highlights the percentiles 2.5 and 97.5 with a thin line. Thick lines are used to highlight the percentiles 25 and 75 while the central markers are used to show the mean values. Red distributions correspond to the null distributions.

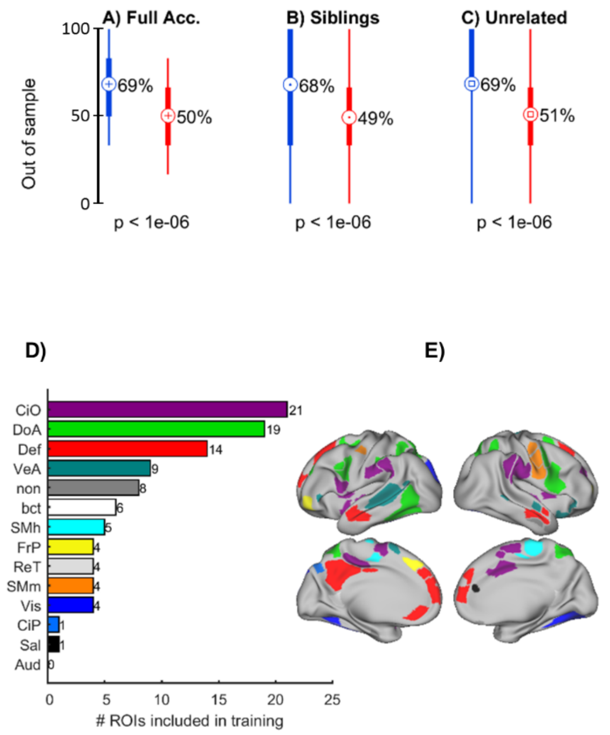

Figure 3-Figure Supplement 6. SVM's out of sample performance for CORRELATIONS, N=1000 per feature set. Mean full accuracy (+), mean accuracy predicting siblings (•), and mean accuracy predicting unrelated population (□). Panel A shows the out of sample performance of the SVM classifiers for correlations as a function of the number of features (x-axis) used for classification. Left-most panel shows the average results as a function of the number of features used for classification. Right-side subpanels show the distributions of accuracy, specificity (accuracy predicting siblings), and specificity (accuracy predicting unrelated participants) compared with the null hypothesis. Panel B show the corresponding results for correlations when the features were selected by functional network, as defined by Gordon (See Figure 2).

A. Correlations, per top features, out of sample accuracy.

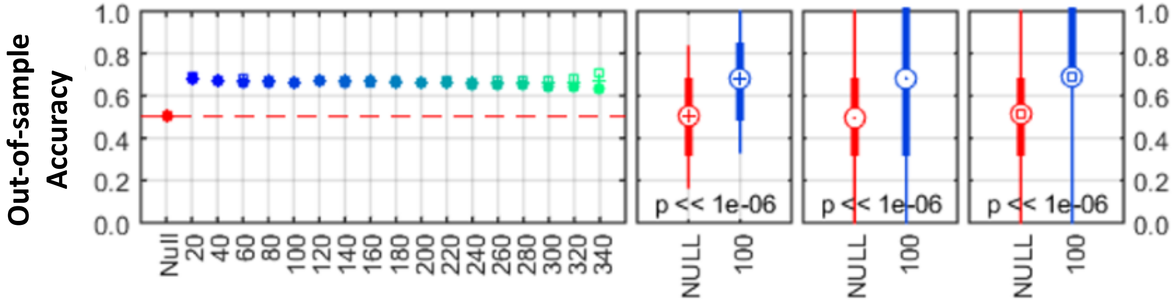

B. Correlations, per network, out of sample accuracy.

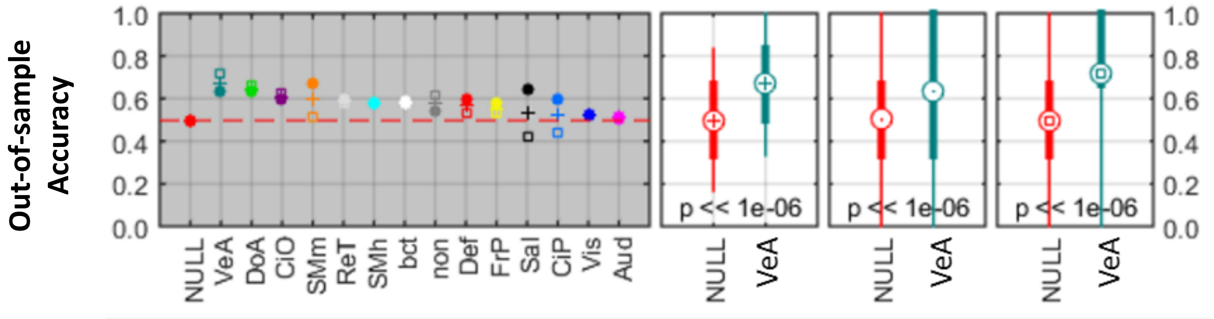

Figure 4-Figure Supplement 1. Classifying siblings vs unrelated populations (N=100 per feature set) using connectotyping and the entire HCP dataset (N=499). Panel A show the mean full accuracy (+), mean accuracy predicting siblings (•), and mean accuracy predicting unrelated population (□) using the correlation coefficient of model-based connectivity matrices (connectotyping) as features. The number of features used for classification were 20, 40, … 340. Analysis was repeated 100 times per feature set. (B-G) shows the distributions of (B) full accuracy, (C) siblings, (D) unrelated, (E) monozygotic, (F) dizygotic, and (G) non-twin sibling pairs for the connectotyping-based SVM classifier.

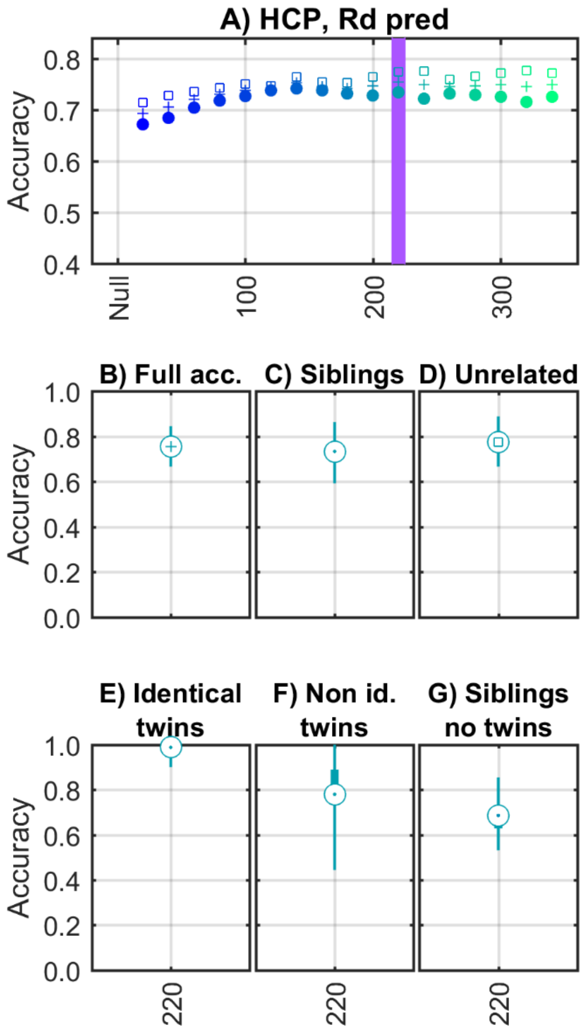

Figure 4-Figure Supplement 2. Classifying siblings vs unrelated populations (N=1000 per feature set), when families with twins and other sibling pairs of the same family were NOT included in the training set (HCP dataset) using a high quality subsample of N=198 scans, using CONNECTOTYPING predicting timecourses. Panel A show the mean full accuracy (+), mean accuracy predicting siblings (•), and mean accuracy predicting unrelated population (□) using the correlation coefficient of model-based connectivity matrices (connectotyping) as features. The number of features used for classification were 20, 40, … 340. Analysis was repeated 1,000 times. (B-G) shows the distributions of (B) full accuracy, (C) siblings, (D) unrelated, (E) monozygotic, (F) dizygotic, and (G) non-twin sibling pairs for the connectotyping-based SVM classifier.

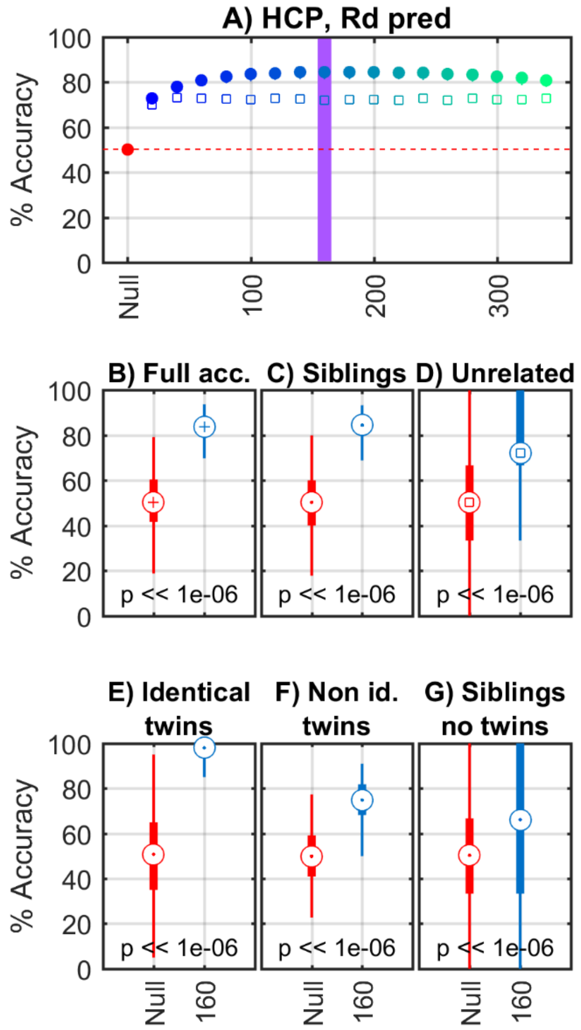

Figure 4-Figure Supplement 3. Classifying siblings vs unrelated populations, when families with twins and other sibling pairs of the same family were NOT included in the training set (HCP dataset) using correlations. Distributions of (A) full accuracy, (B) siblings, (C) unrelated, (D) monozygotic, (E) dizygotic, and (F) non-twin sibling pairs for the correlations-based SVM classifier. (G) The consensus' distribution of ROI's per functional network used in the classifier. (H) The location of such ROIs in the surface of the brain. Each distribution highlights the percentiles 2.5 and 97.5 with a thin line. Thick lines are used to highlight the percentiles 25 and 75 while the central markers are used to show the mean values. Red distributions correspond to the null distributions.

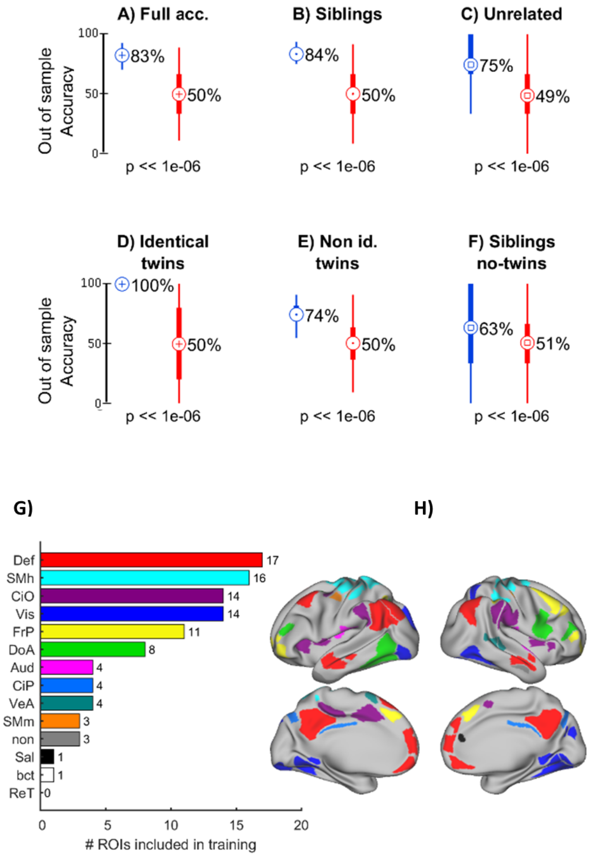

Figure 4-Figure Supplement 4. Classifying siblings vs unrelated populations (N=1000 per feature set), when families with twins and other sibling pairs of the same family were NOT included in the training set (HCP dataset) using a high quality subsample of N=198 scans, using traditional CORRELATIONS to characterize brain connectivity. Panel A show the mean full accuracy (+), mean accuracy predicting siblings (•), and mean accuracy predicting unrelated population (□) using the correlation coefficient of model-based connectivity matrices (connectotyping) as features. The number of features used for classification were 20, 40, … 340. Analysis was repeated 1,000 times. (B-G) shows the distributions of (B) full accuracy, (C) siblings, (D) unrelated, (E) monozygotic, (F) dizygotic, and (G) non-twin sibling pairs for the connectotyping-based SVM classifier.

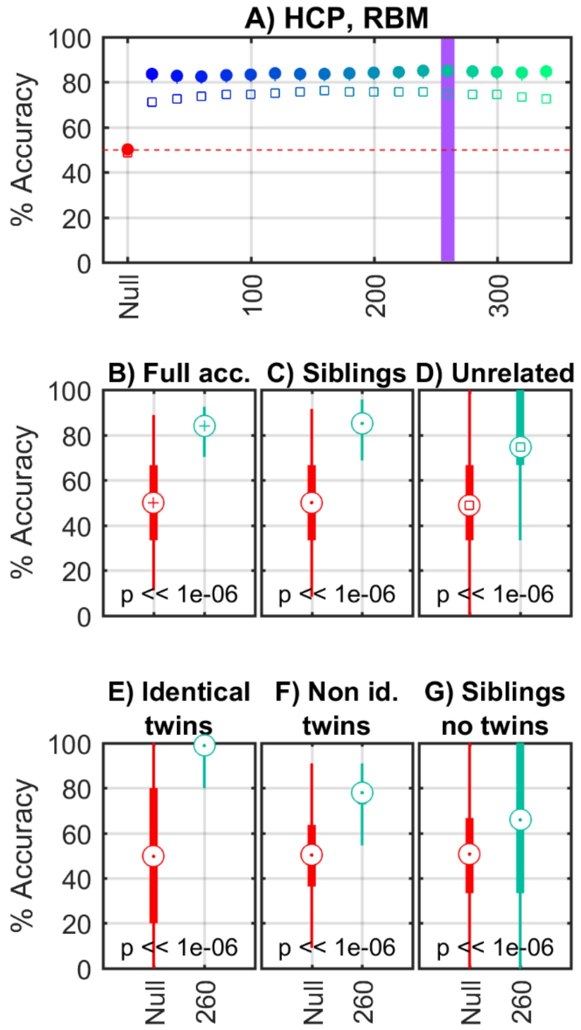

Figure 5-Figure Supplement 1. Dataset 1 predicting dataset 2 using connectotyping. Distributions of out-of-sample accuracies (N=1000 per feature set) when classifiers were trained in one dataset (OHSU, ie youth, or HCP, ie adults) and tested in the other dataset.

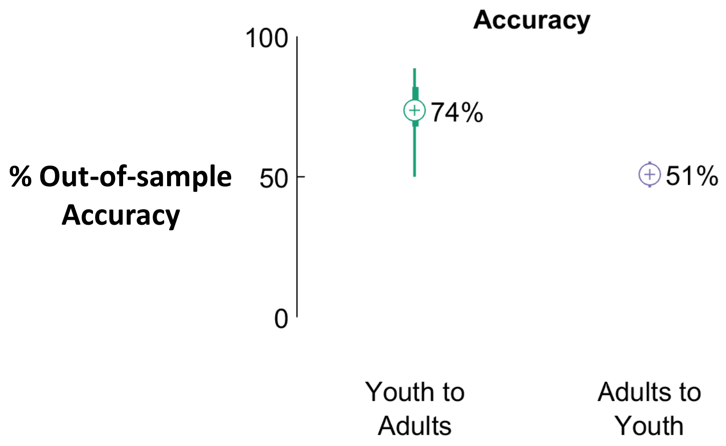

Figure 5-Figure Supplement 2. Dataset 1 predicting dataset 2 using traditional correlations. Distributions of out-of-sample accuracies (N=1000 per feature set) when classifiers were trained in one dataset (OHSU, ie youth, or HCP, ie adults) and tested in the other dataset.

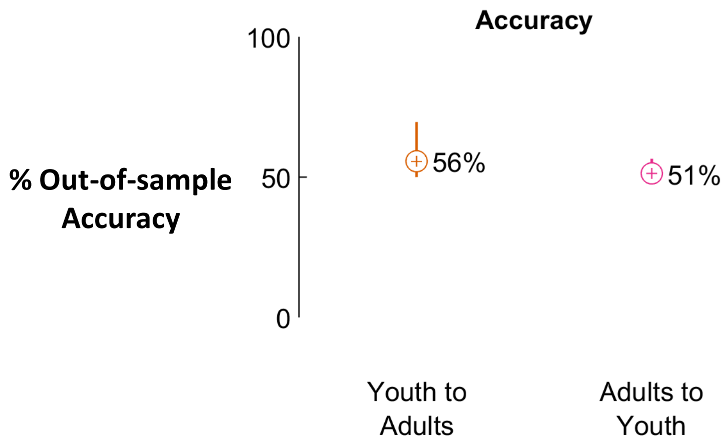

Figure 6-Figure Supplement 1. Out of sample performance of classifiers using connectotyping (green) and different anatomical features (brown) to classify kinship in adults when classifiers were trained using data from an independent dataset of youths. Green traces correspond to the results of the classification using connectotyping, as shown in figures 5 and 6. Same classification procedure was repeated using cortical thickness and sulcal depth as features (after removing the effect of head size by normalization of regression), but using the top 100 more distinct features according to connectotyping (light brown, labeled as "Comb" to indicate "Combined"). Dark brown lines show the performance of the classifiers when features and feature selection was based on anatomical features.

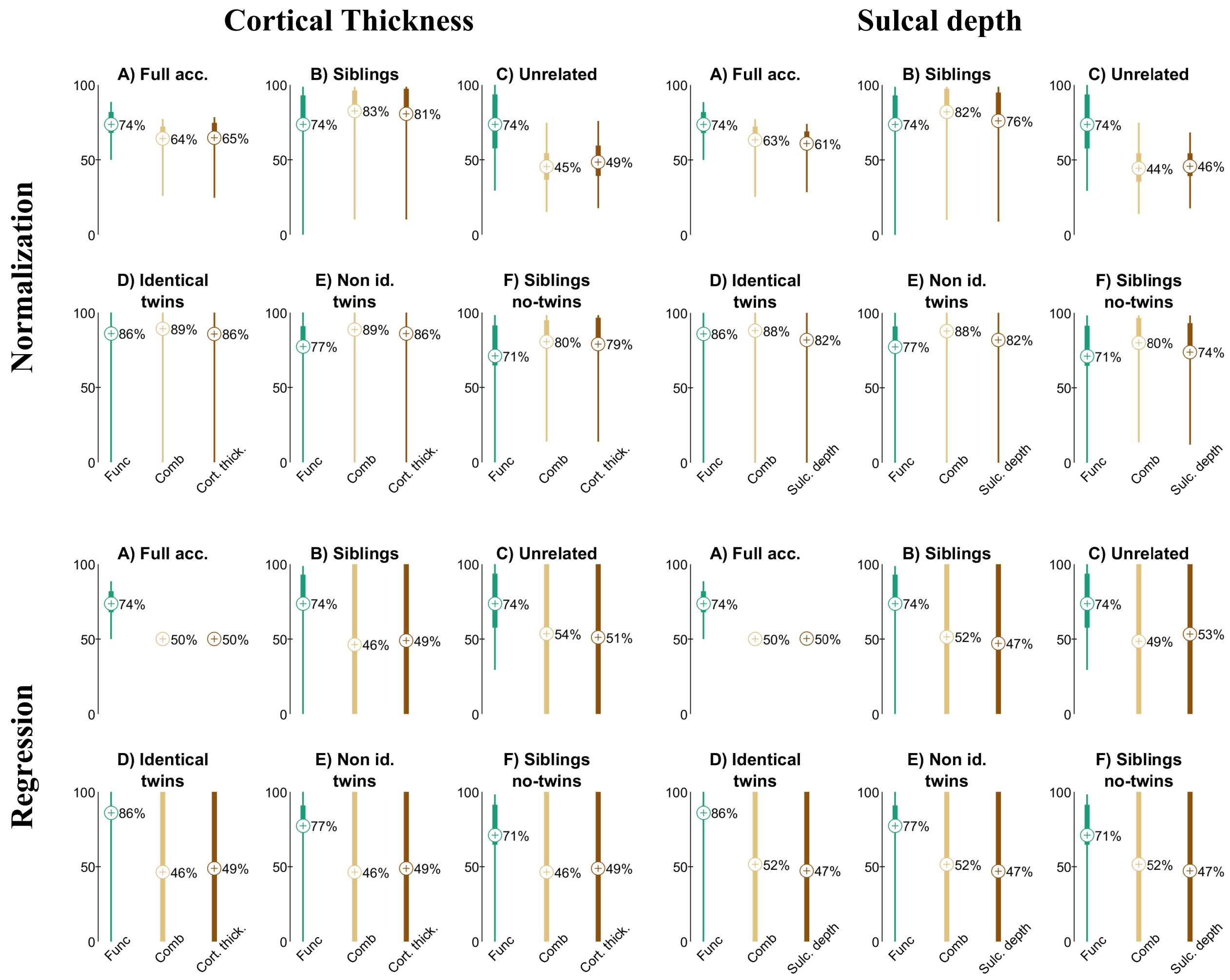

Figure 7-Figure Supplement 1. Summary of heritability (circle) and shared environment (plus sign) analyses conducted on 198 (red) and 499 (blue) participants (connectotyping).

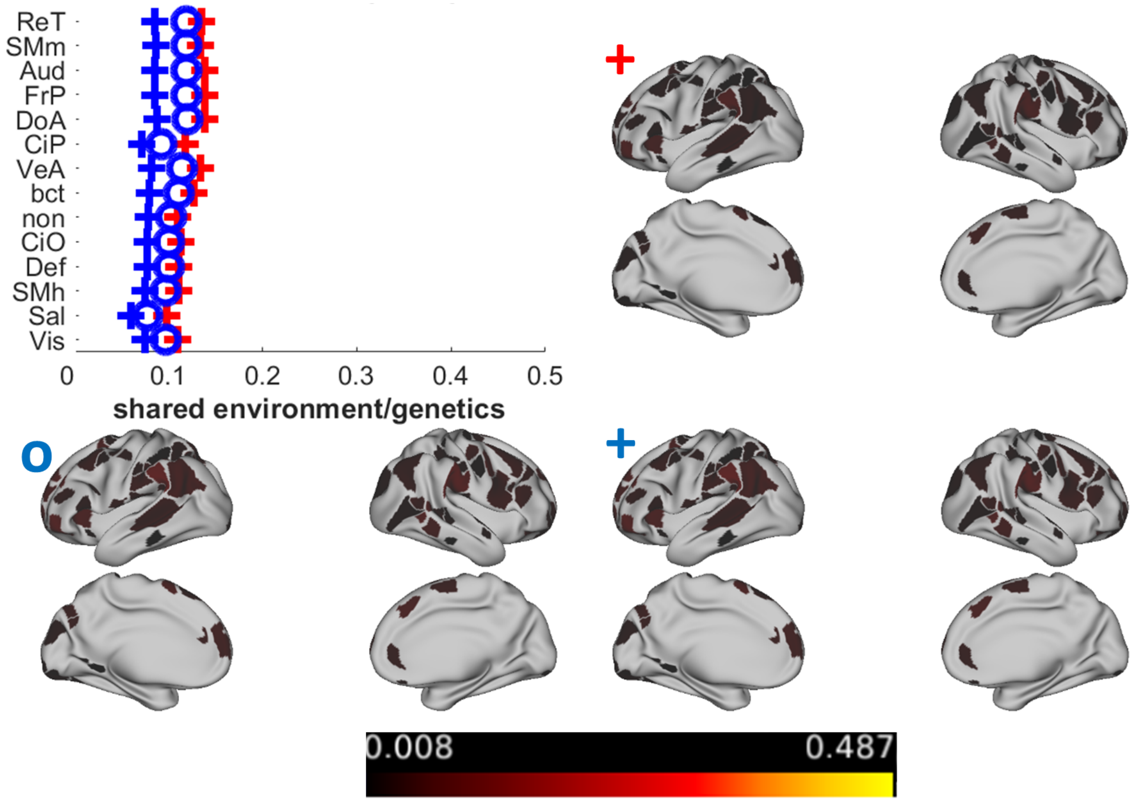

\+ - shared environment 198 participants
\+ - shared environment 499 pariticipants
o – shared genetics 499 participants

Figure 7-Figure Supplement 2. Heritability traditional functional connectivity correlations

(7A) The Spatial correlation's heritability of the top 100 regions used in the SVM classification for Traditional correlations (see: Figure 3-Figure Supplement 5). (Panel B) The spatial correlation heritability for each network. Networks are sorted from most to least heritable, and the bar color matches the networks shown in figure 2.

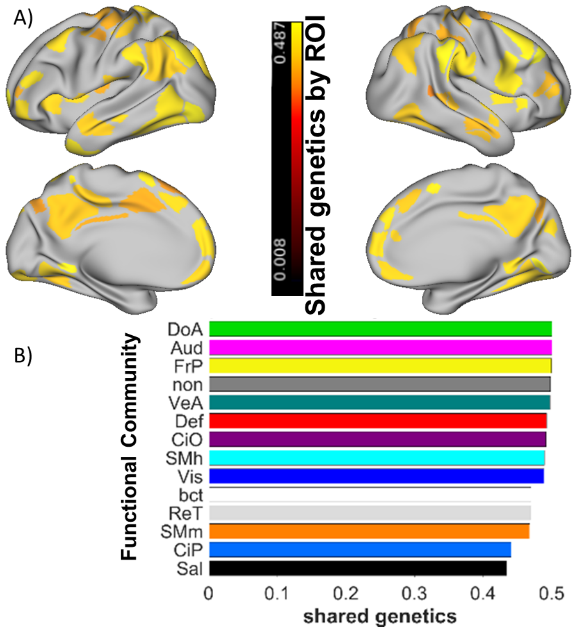

Figure 7-Figure Supplement 3. Summary of heritability (circle) and shared environment (plus sign) analyses conducted on 198 (red) and 499 (blue) participants for spatial correlations. Traditional heritability (black circle) and shared environment (black plus sign) on traditional correlations are also shown.

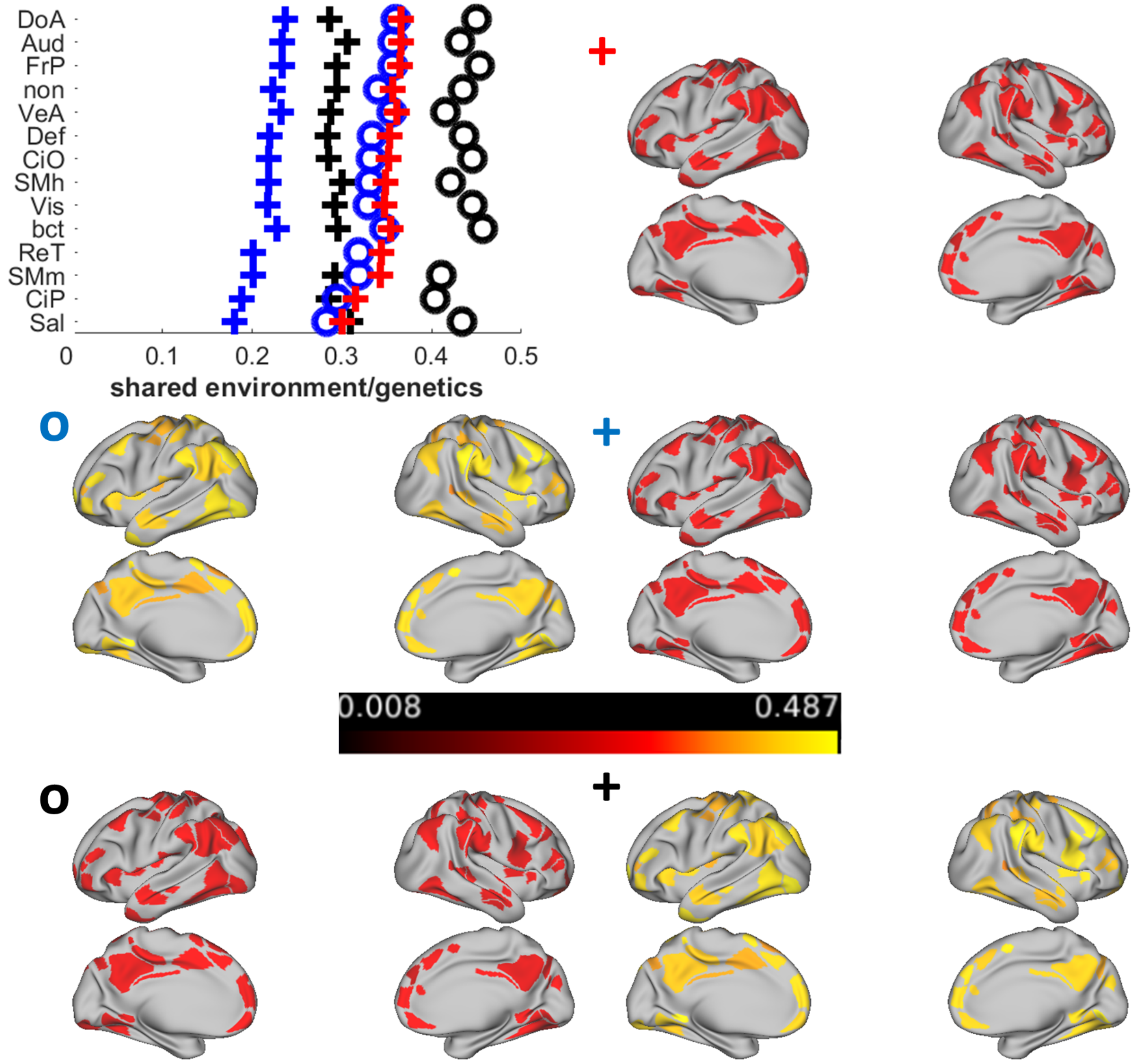

+ - shared environment 198 participants
+ - shared environment 499 pariticipants
+ - GLMM shared environment 198 participants for top 100 ROIs
o – shared genetics 499 participants
o – GLMM shared genetics 198 participants for top 100 ROIs

Supplemental tables.

Table S1. Siblings included in the study

Family	Siblings *per* family	Scans *per* sibling		Correlations of connectivity matrices *per* family	Predictions *per* family
**1**	2	2	1	2	4
**2**	2	1	1	1	2
**3**	2	1	1	1	2
**4**	2	2	2	4	8
**5**	2	1	3	3	6
**6**	2	1	1	1	2
**7**	2	1	2	2	4
**8**	2	1	1	1	2
**9**	2	1	1	1	2
**10**	2	1	1	1	2
**11**	2	1	1	1	2
**12**	2	1	1	1	2
**13**	2	1	1	1	2
**14**	2	1	1	1	2
**15**	2	1	1	1	2
**16**	2	1	1	1	2
		**Total**		**23**	**46**

Table S2. Ranking of the individuals ROIs to differentiate between siblings and unrelated.

Index	ROI name	Network	Number of grayordinates
1	155_L_DorsalAttn	L DoA	72
2	200_R_Default	R Def	194
3	240_R_FrontoParietal	R FrP	104
4	144_L_None	L non	74
5	219_R_CinguloOperc	R CiO	218
6	PALLIDUM_RIGHT	Subct	260
7	75_L_VentralAttn	L VeA	104
8	74_L_DorsalAttn	L DoA	86
9	153_L_CinguloOperc	L CiO	84
10	60_L_VentralAttn	L VeA	49
11	103_L_CinguloOperc	L CiO	95
12	295_R_RetrosplenialTemporal	R ReT	87
13	110_L_DorsalAttn	L DoA	85
14	113_L_DorsalAttn	L DoA	42
15	4_L_Default	L Def	163
16	25_L_Default	L Def	84
17	146_L_Default	L Def	99
18	165_R_Default	R Def	161
19	89_L_CinguloParietal	L CiP	54
20	281_R_None	R non	76
21	122_L_None	L non	57
22	44_L_Default	L Def	223
23	119_L_None	L non	42
24	157_L_Default	L Def	147
25	106_L_DorsalAttn	L DoA	239
26	222_R_VentralAttn	R VeA	58
27	277_R_FrontoParietal	R FrP	104
28	290_R_Default	R Def	223
29	18_L_None	L non	62
30	79_L_VentralAttn	L VeA	50
31	158_L_VentralAttn	L VeA	70
32	170_R_FrontoParietal	R FrP	116
33	331_R_Default	R Def	30
34	262_R_DorsalAttn	R DoA	130
35	51_L_DorsalAttn	L DoA	231
36	HIPPOCAMPUS_LEFT	Subct	764
37	194_R_SMhand	R SMh	34
38	BRAIN_STEM	Subct	3472

Index	ROI name	Network	Number of grayordinates
**39**	230_R_Auditory	R Aud	57
**40**	37_L_SMhand	L SMh	70
**41**	174_R_RetrosplenialTemporal	R ReT	257
**42**	23_L_VentralAttn	L VeA	84
**43**	296_R_None	R non	77
**44**	14_L_RetrosplenialTemporal	L ReT	128
**45**	333_R_VentralAttn	R VeA	43
**46**	332_R_VentralAttn	R VeA	44
**47**	154_L_Default	L Def	59
**48**	12_L_CinguloParietal	L CiP	93
**49**	63_L_CinguloOperc	L CiO	226
**50**	220_R_Default	R Def	444
**51**	225_R_Default	R Def	85
**52**	AMYGDALA_LEFT	Subct	315
**53**	PUTAMEN_RIGHT	Subct	1010
**54**	322_R_Default	R Def	188
**55**	101_L_CinguloOperc	L CiO	85
**56**	2_L_SMhand	L SMh	275
**57**	164_R_SMmouth	R SMm	132
**58**	198_R_CinguloOperc	R CiO	81
**59**	50_L_SMhand	L SMh	119
**60**	108_L_FrontoParietal	L FrP	43
**61**	316_R_Default	R Def	55
**62**	242_R_VentralAttn	R VeA	132
**63**	83_L_Salience	L Sal	32
**64**	80_L_VentralAttn	L VeA	236
**65**	180_R_CinguloOperc	R CiO	459
**66**	112_L_CinguloOperc	L CiO	159
**67**	283_R_None	R non	106
**68**	325_R_Default	R Def	75
**69**	CEREBELLUM_LEFT	Subct	8709
**70**	239_R_Auditory	R Aud	82
**71**	238_R_CinguloOperc	R CiO	134
**72**	156_L_Default	L Def	54
**73**	247_R_Salience	R Sal	62
**74**	107_L_DorsalAttn	L DoA	49
**75**	HIPPOCAMPUS_RIGHT	Subct	795
**76**	7_L_FrontoParietal	L FrP	35
**77**	317_R_CinguloOperc	R CiO	48

Index	ROI name	Network	Number of grayordinates
**78**	320_R_FrontoParietal	R FrP	44
**79**	160_L_Auditory	L Aud	122
**80**	123_L_None	L non	82
**81**	62_L_VentralAttn	L VeA	645
**82**	136_L_Visual	L Vis	80
**83**	315_R_Default	R Def	115
**84**	297_R_None	R non	44
**85**	274_R_CinguloOperc	R CiO	114
**86**	217_R_SMhand	R SMh	91
**87**	162_R_Default	R Def	674
**88**	105_L_CinguloOperc	L CiO	208
**89**	311_R_Visual	R Vis	35
**90**	147_L_CinguloOperc	L CiO	30
**91**	275_R_DorsalAttn	R DoA	405
**92**	96_L_FrontoParietal	L FrP	181
**93**	PALLIDUM_LEFT	Subct	297
**94**	205_R_SMhand	R SMh	103
**95**	142_L_None	L non	71
**96**	201_R_SMhand	R SMh	140
**97**	81_L_CinguloOperc	L CiO	45
**98**	128_L_None	L non	137
**99**	324_R_Default	R Def	66
**100**	1_L_Default	L Def	715
**101**	182_R_FrontoParietal	R FrP	97
**102**	236_R_DorsalAttn	R DoA	104
**103**	54_L_SMhand	L SMh	70
**104**	286_R_None	R non	35
**105**	139_L_Visual	L Vis	148
**106**	30_L_SMhand	L SMh	35
**107**	39_L_SMmouth	L SMm	63
**108**	93_L_CinguloParietal	L CiP	118
**109**	184_R_Default	R Def	132
**110**	280_R_None	R non	66
**111**	138_L_Visual	L Vis	57
**112**	292_R_None	R non	246
**113**	127_L_Default	L Def	295
**114**	73_L_None	L non	129
**115**	78_L_FrontoParietal	L FrP	145
**116**	109_L_FrontoParietal	L FrP	151

Index	ROI name	Network	Number of grayordinates
**117**	234_R_CinguloOperc	R CiO	184
**118**	221_R_VentralAttn	R VeA	61
**119**	DIENCEPHALON_VENTRAL_RIGHT	Subct	712
**120**	159_L_None	L non	70
**121**	266_R_DorsalAttn	R DoA	42
**122**	173_R_CinguloParietal	R CiP	90
**123**	199_R_DorsalAttn	R DoA	67
**124**	150_L_Default	L Def	280
**125**	71_L_CinguloOperc	L CiO	152
**126**	43_L_DorsalAttn	L DoA	56
**127**	254_R_CinguloParietal	R CiP	159
**128**	111_L_CinguloOperc	L CiO	102
**129**	91_L_DorsalAttn	L DoA	198
**130**	270_R_SMhand	R SMh	117
**131**	212_R_SMmouth	R SMm	361
**132**	21_L_CinguloOperc	L CiO	326
**133**	318_R_CinguloOperc	R CiO	164
**134**	287_R_None	R non	89
**135**	114_L_Default	L Def	41
**136**	181_R_CinguloOperc	R CiO	44
**137**	90_L_Visual	L Vis	83
**138**	214_R_SMhand	R SMh	128
**139**	243_R_VentralAttn	R VeA	123
**140**	92_L_DorsalAttn	L DoA	52
**141**	THALAMUS_LEFT	Subct	1288
**142**	267_R_Visual	R Vis	92
**143**	61_L_VentralAttn	L VeA	42
**144**	206_R_SMhand	R SMh	43
**145**	314_R_None	R non	37
**146**	64_L_Auditory	L Aud	46
**147**	223_R_CinguloOperc	R CiO	829
**148**	ACCUMBENS_LEFT	Subct	135
**149**	258_R_Visual	R Vis	81
**150**	185_R_CinguloOperc	R CiO	250
**151**	291_R_None	R non	48
**152**	241_R_VentralAttn	R VeA	32
**153**	276_R_FrontoParietal	R FrP	48
**154**	45_L_SMhand	L SMh	127
**155**	232_R_Auditory	R Aud	36

Index	ROI name	Network	Number of grayordinates
**156**	117_L_Default	L Def	144
**157**	172_R_None	R non	98
**158**	9_L_FrontoParietal	L FrP	49
**159**	121_L_None	L non	49
**160**	268_R_Auditory	R Aud	58
**161**	231_R_VentralAttn	R VeA	96
**162**	CEREBELLUM_RIGHT	Subct	9144
**163**	24_L_FrontoParietal	L FrP	111
**164**	140_L_Visual	L Vis	143
**165**	72_L_CinguloOperc	L CiO	179
**166**	132_L_Visual	L Vis	63
**167**	305_R_None	R non	33
**168**	235_R_CinguloOperc	R CiO	163
**169**	265_R_Visual	R Vis	376
**170**	319_R_FrontoParietal	R FrP	89
**171**	272_R_FrontoParietal	R FrP	99
**172**	DIENCEPHALON_VENTRAL_LEFT	Subct	706
**173**	328_R_FrontoParietal	R FrP	77
**174**	327_R_FrontoParietal	R FrP	171
**175**	98_L_Visual	L Vis	84
**176**	293_R_Visual	R Vis	32
**177**	207_R_SMhand	R SMh	85
**178**	168_R_FrontoParietal	R FrP	70
**179**	288_R_None	R non	22
**180**	118_L_None	L non	47
**181**	228_R_VentralAttn	R VeA	56
**182**	THALAMUS_RIGHT	Subct	1248
**183**	31_L_SMhand	L SMh	153
**184**	104_L_Auditory	L Aud	136
**185**	183_R_Salience	R Sal	40
**186**	49_L_DorsalAttn	L DoA	60
**187**	259_R_Default	R Def	350
**188**	47_L_SMhand	L SMh	183
**189**	56_L_SMhand	L SMh	240
**190**	278_R_Default	R Def	74
**191**	19_L_None	L non	35
**192**	53_L_SMmouth	L SMm	405
**193**	84_L_CinguloOperc	L CiO	244
**194**	13_L_RetrosplenialTemporal	L ReT	134

Index	ROI name	Network	Number of grayordinates
**195**	279_R_Default	R Def	155
**196**	177_R_Visual	R Vis	86
**197**	129_L_None	L non	161
**198**	52_L_DorsalAttn	L DoA	160
**199**	196_R_CinguloOperc	R CiO	126
**200**	209_R_SMhand	R SMh	80
**201**	245_R_CinguloOperc	R CiO	65
**202**	229_R_VentralAttn	R VeA	109
**203**	11_L_None	L non	52
**204**	249_R_CinguloOperc	R CiO	207
**205**	66_L_Auditory	L Aud	155
**206**	76_L_CinguloOperc	L CiO	224
**207**	145_L_Default	L Def	97
**208**	330_R_Auditory	R Aud	142
**209**	257_R_Default	R Def	39
**210**	82_L_CinguloOperc	L CiO	35
**211**	33_L_SMhand	L SMh	30
**212**	289_R_None	R non	34
**213**	273_R_FrontoParietal	R FrP	167
**214**	213_R_SMhand	R SMh	281
**215**	312_R_None	R non	83
**216**	135_L_None	L non	73
**217**	326_R_Default	R Def	46
**218**	22_L_CinguloOperc	L CiO	306
**219**	299_R_Visual	R Vis	152
**220**	252_R_DorsalAttn	R DoA	243
**221**	143_L_RetrosplenialTemporal	L ReT	51
**222**	26_L_Default	L Def	43
**223**	263_R_Visual	R Vis	526
**224**	77_L_Auditory	L Aud	112
**225**	3_L_SMmouth	L SMm	170
**226**	302_R_None	R non	34
**227**	204_R_SMhand	R SMh	35
**228**	PUTAMEN_LEFT	Subct	1060
**229**	169_R_Visual	R Vis	38
**230**	125_L_None	L non	31
**231**	163_R_SMhand	R SMh	304
**232**	57_L_SMhand	L SMh	104
**233**	34_L_CinguloOperc	L CiO	137

Index	ROI name	Network	Number of grayordinates
**234**	264_R_Visual	R Vis	235
**235**	179_R_None	R non	45
**236**	269_R_Auditory	R Aud	84
**237**	190_R_SMhand	R SMh	196
**238**	AMYGDALA_RIGHT	Subct	332
**239**	100_L_DorsalAttn	L DoA	538
**240**	244_R_Auditory	R Aud	39
**241**	67_L_Auditory	L Aud	59
**242**	253_R_DorsalAttn	R DoA	138
**243**	151_L_Default	L Def	47
**244**	186_R_Default	R Def	30
**245**	260_R_FrontoParietal	R FrP	52
**246**	313_R_RetrosplenialTemporal	R ReT	34
**247**	102_L_Auditory	L Aud	61
**248**	166_R_Visual	R Vis	239
**249**	167_R_FrontoParietal	R FrP	143
**250**	59_L_SMmouth	L SMm	46
**251**	40_L_CinguloOperc	L CiO	45
**252**	29_L_Salience	L Sal	38
**253**	28_L_CinguloOperc	L CiO	31
**254**	116_L_Default	L Def	183
**255**	97_L_Visual	L Vis	255
**256**	178_R_None	R non	57
**257**	202_R_SMhand	R SMh	72
**258**	36_L_SMhand	L SMh	188
**259**	227_R_Auditory	R Aud	143
**260**	187_R_CinguloOperc	R CiO	45
**261**	323_R_Default	R Def	34
**262**	99_L_Visual	L Vis	244
**263**	68_L_Auditory	L Aud	76
**264**	148_L_FrontoParietal	L FrP	68
**265**	197_R_SMmouth	R SMm	105
**266**	85_L_VentralAttn	L VeA	54
**267**	271_R_DorsalAttn	R DoA	115
**268**	188_R_CinguloOperc	R CiO	66
**269**	CAUDATE_LEFT	Subct	728
**270**	41_L_DorsalAttn	L DoA	168
**271**	203_R_DorsalAttn	R DoA	128
**272**	133_L_None	L non	70

Index	ROI name	Network	Number of grayordinates
**273**	321_R_Default	R Def	113
**274**	124_L_None	L non	34
**275**	126_L_Default	L Def	51
**276**	195_R_SMhand	R SMh	73
**277**	CAUDATE_RIGHT	Subct	755
**278**	38_L_SMhand	L SMh	243
**279**	282_R_None	R non	52
**280**	134_L_None	L non	41
**281**	216_R_SMhand	R SMh	270
**282**	310_R_Visual	R Vis	59
**283**	86_L_VentralAttn	L VeA	56
**284**	246_R_CinguloOperc	R CiO	60
**285**	6_L_Default	L Def	887
**286**	261_R_FrontoParietal	R FrP	37
**287**	137_L_Visual	L Vis	181
**288**	284_R_None	R non	79
**289**	171_R_Auditory	R Aud	129
**290**	46_L_SMhand	L SMh	52
**291**	208_R_DorsalAttn	R DoA	69
**292**	141_L_Visual	L Vis	303
**293**	215_R_SMhand	R SMh	42
**294**	88_L_DorsalAttn	L DoA	139
**295**	192_R_CinguloOperc	R CiO	69
**296**	55_L_DorsalAttn	L DoA	120
**297**	193_R_SMhand	R SMh	111
**298**	5_L_Visual	L Vis	389
**299**	94_L_Default	L Def	247
**300**	152_L_Default	L Def	72
**301**	48_L_SMhand	L SMh	72
**302**	ACCUMBENS_RIGHT	Subct	140
**303**	218_R_SMmouth	R SMm	54
**304**	27_L_CinguloOperc	L CiO	138
**305**	304_R_None	R non	79
**306**	20_L_Visual	L Vis	71
**307**	87_L_DorsalAttn	L DoA	315
**308**	251_R_Visual	R Vis	179
**309**	131_L_Visual	L Vis	117
**310**	65_L_Auditory	L Aud	41
**311**	175_R_Visual	R Vis	665

Index	ROI name	Network	Number of grayordinates
**312**	256_R_Visual	R Vis	31
**313**	69_L_Auditory	L Aud	59
**314**	15_L_Visual	L Vis	636
**315**	248_R_CinguloOperc	R CiO	42
**316**	233_R_Auditory	R Aud	117
**317**	294_R_RetrosplenialTemporal	R ReT	36
**318**	250_R_DorsalAttn	R DoA	38
**319**	70_L_Auditory	L Aud	33
**320**	191_R_SMhand	R SMh	256
**321**	8_L_Visual	L Vis	177
**322**	301_R_None	R non	68
**323**	130_L_RetrosplenialTemporal	L ReT	145
**324**	298_R_Visual	R Vis	407
**325**	176_R_Visual	R Vis	139
**326**	115_L_None	L non	34
**327**	307_R_Visual	R Vis	100
**328**	285_R_None	R non	14
**329**	149_L_FrontoParietal	L FrP	48
**330**	224_R_Auditory	R Aud	135
**331**	300_R_None	R non	73
**332**	255_R_Visual	R Vis	67
**333**	226_R_VentralAttn	R VeA	55
**334**	120_L_None	L non	108
**335**	32_L_SMhand	L SMh	58
**336**	309_R_Visual	R Vis	66
**337**	10_L_Auditory	L Aud	106
**338**	42_L_DorsalAttn	L DoA	48
**339**	211_R_DorsalAttn	R DoA	181
**340**	161_L_VentralAttn	L VeA	37
**341**	306_R_None	R non	52
**342**	210_R_SMhand	R SMh	35
**343**	58_L_SMhand	L SMh	119
**344**	189_R_DorsalAttn	R DoA	258
**345**	329_R_Auditory	R Aud	10
**346**	16_L_Visual	L Vis	39
**347**	303_R_None	R non	70
**348**	95_L_DorsalAttn	L DoA	45
**349**	35_L_SMhand	L SMh	134
**350**	308_R_Visual	R Vis	84

Index	ROI name	Network	Number of grayordinates
**351**	237_R_VentralAttn	R VeA	60
**352**	17_L_Visual	L Vis	14

Table S3. Mapping of the top 11 unclassified ROIs from Gordon into Yeo, based on grayordinates. The grayordinates from each ROI were map into each one of the 7 networks proposed by Yeo

Relative importance	Gordon ROI name	# grayordinates in Gordon	Yeo-Limbic	Yeo-FrP	Yeo-Def	Yeo-No assignment	Winner take all
**4**	144_L_None	74	8	0	**35**	31	Def
**20**	281_R_None	76	0	13	**63**	0	Def
**21**	122_L_None	57	**57**	0	0	0	Limbic
**23**	119_L_None	42	0	15	**27**	0	Def
**29**	18_L_None	62	0	0	1	**61**	Non
**43**	296_R_None	77	**46**	0	24	7	Limbic
**67**	283_R_None	106	**85**	21	0	0	Limbic
**80**	123_L_None	82	**82**	0	0	0	Limbic
**84**	297_R_None	44	**44**	0	0	0	Limbic
**95**	142_L_None	71	**52**	0	1	18	Limbic
**98**	128_L_None	137	**80**	0	57	0	Limbic

Table S4. Sibling status for participants form the Human Connectome Project[1].

# families	Descendants per family	Sibling type			Total
		Identical twins	Non identical twins	Siblings no twins	
**70**	1	0	0	0	**0**
**49**	2	5	10	34	**49**
**10**	3	5	1	24	**30**
	**Total**	**10**	**11**	**58**	**79**

[1] Open access and restricted data from the Washington University in Saint Louis-University of Minnesota (WU-Minn) HCP consortium "500 Subjects release" (June 2014) was generously provided after registration and agreement of the Open Access Data Use Terms and Restricted Data Use Terms

Table S5. Partitions used for classifying siblings from unrelated populations using HCP data and SVM.

**Method**	**Identical twins**		**Non-identical twins**		**Siblings-no-twins**		**Unrelated**	
	In-sample	Out-of-sample	In-sample	Out-of-sample	In-sample	Out-of-sample	In-sample	Out-of-sample
**Connectotyping**	16	4	18	4	104	12	138	20
**Correlations of connectivity matrices**	6	4	7	4	54	4	67	12

Supplemental References.

Benjamini, Y., & Hochberg, Y. (1995). Controlling the False Discovery Rate: a Practical and Powerful Approach to Multiple Testing. *J.R. Statist, Soc, B*, *57*(1), 289–300.

Dale, A.M., Fischl, B. & Sereno, M.I., 1999. Cortical surface-based analysis. I. Segmentation and surface reconstruction. *NeuroImage*, 9(2), pp.179–94.

Desikan, R.S. et al., 2006. An automated labeling system for subdividing the human cerebral cortex on MRI scans into gyral based regions of interest. *NeuroImage*, 31(3), pp.968–80.

Dosenbach, N.U.F. et al., 2010. Prediction of individual brain maturity using fMRI. *Science (New York, N.Y.)*, 329(5997), pp.1358–61.

Fischl, B. & Dale, A.M., 2000. Measuring the thickness of the human cerebral cortex from magnetic resonance images. *Proceedings of the National Academy of Sciences of the United States of America*, 97(20), pp.11050–5.

Glahn, D. C., Winkler, a M., Kochunov, P., Almasy, L., Duggirala, R., Carless, M. a, … Blangero, J. (2010). Genetic control over the resting brain. *Proceedings of the National Academy of Sciences of the United States of America*, *107*(3), 1223–1228. http://doi.org/10.1073/pnas.0909969107

Greve, D.N. & Fischl, B., 2009. Accurate and robust brain image alignment using boundary-based registration. *NeuroImage*, 48(1), pp.63–72.

Jenkinson, M. et al., 2012. FSL. *NeuroImage*, 62(2), pp.782–90.

Smith, S.M. et al., 2004. Advances in functional and structural MR image analysis and implementation as FSL. *NeuroImage*, 23 Suppl 1, pp.S208–19.

Visscher, P. M., & Goddard, M. E. (2015). A General Unified Framework to Assess the Sampling Variance of Heritability Estimates Using Pedigree or Marker-based Relationships. *Genetics*, *199*(January), 223–232.

Woolrich, M.W. et al., 2009. Bayesian analysis of neuroimaging data in FSL. *NeuroImage*, 45(1 Suppl), pp.S173–86.
